# Negative Consequences of Removing GLP‐1 RA Obesity Coverage: A Cross‐Sectional Cohort Comparison Study

**DOI:** 10.1002/osp4.70123

**Published:** 2026-03-03

**Authors:** Jackson M. Francis, Deepali K. Ernest, Aparajita Chandrasekhar, Grant Herrington, Chellse Gazda, Luyu Xie, M. Sunil Mathew, Marianne Olaniran, Sarah E. Messiah, Jaime P. Almandoz

**Affiliations:** ^1^ Department of Epidemiology, Peter O'Donnell School of Public Health, University of Texas Southwestern Medical Center Dallas Texas USA; ^2^ Department of Epidemiology, School of Public Health, University of Texas Health Science Center at Houston Houston Texas USA; ^3^ Department of Internal Medicine Division of Endocrinology University of Texas Southwestern Medical Center Dallas Texas USA; ^4^ Department of Epidemiology, School of Public Health, University of Texas Health Science Center at Houston Dallas Texas USA; ^5^ Department of Pediatrics University of Texas Southwestern Medical Center Dallas Texas USA; ^6^ Children's Health System of Texas Dallas Texas USA

**Keywords:** employee morale, GLP‐1 RA, insurance coverage

## Abstract

**Objective:**

GLP‐1 receptor agonist (GLP‐1 RA) coverage decisions for obesity have major health and economic implications. This study examined how loss of GLP‐1 coverage affected employee perceptions of their employer and workforce stability.

**Methods:**

This study analyzed data from employees of a healthcare system that discontinued GLP‐1 obesity medication coverage. Outcomes were evaluated using chi‐square/Fisher's exact tests and multivariable logistic regression.

**Results:**

Two hundred forty‐seven adults prescribed GLP‐1 for obesity who lost coverage (mean age 49.27 years—SD: 9.89, BMI 32.85 kg/m^2^—SD: 8.49); 90.28% were female, 40.08% non‐Hispanic White, 30.36% non‐Hispanic Black, 19.84% Hispanic, and 9.72% Other. Following loss, 80.57% reported worsened employer perceptions, 17.81% considered changing jobs, 49.39% felt devalued, and 64.38% believed their employer didn't value their health. Burnout was common (88.67% past year; 65.19% past month). NHB and Hispanic employees were less likely than NHW to report worsened employer relationships (aOR = 0.29 and 0.34), while those with incomes ≥ $150,000 were 2.5x as likely to report negative impact (aOR = 2.57).

**Conclusion:**

Loss of GLP‐1 coverage was linked to reduced employee satisfaction, increased burnout, and greater turnover risk. Lower reported impact among NHB and Hispanic employees may reflect disparities in perceived job mobility. These consequences may undermine workforce stability and offset cost savings that motivate coverage restrictions.

## Graphical abstract

This study examined the impact of discontinuing GLP‐1 obesity medication coverage among 247 healthcare employees. Loss of coverage was associated with worsened perceptions of the employer, feelings of being devalued, high levels of burnout, and consideration of job change, particularly among higher‐income employees. These findings suggest that restricting GLP‐1 coverage may harm workforce satisfaction and stability, potentially offsetting financial savings.

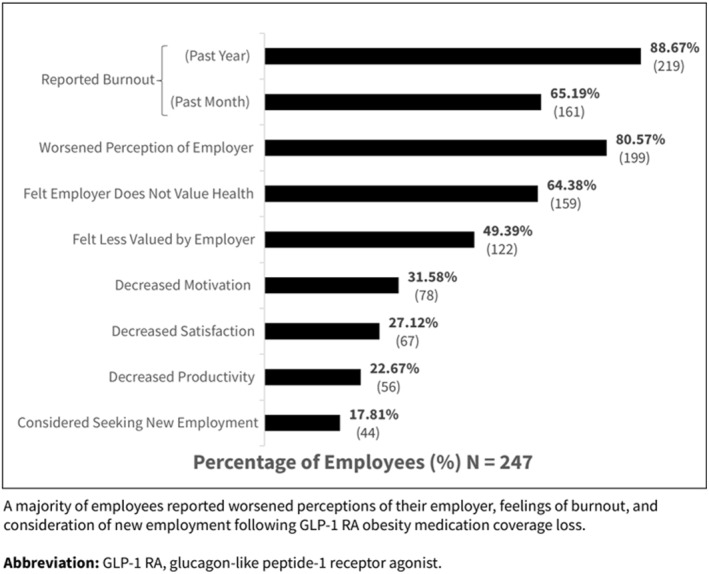

## 1 Introduction

Rising healthcare costs have prompted several employer‐sponsored insurance plans to discontinue or restrict coverage for obesity medications, such as glucagon‐like peptide‐1 receptor agonists (GLP‐1 RAs) [1]. While intended as cost‐saving measures, these restrictions may have a negative impact on employee satisfaction, retention, productivity, and workplace dynamics.

Effective treatments, including GLP‐1 RAs, have transformed obesity care by providing a non‐surgical alternative that promotes clinically significant weight loss and improvements in obesity‐related complications [2]. As of 2024, approximately one in eight U.S. adults has used a GLP‐1 RA, reflecting substantial demand [3]. Obesity and its associated complications drive significant increases in healthcare expenditures and productivity losses [4]. Evidence suggests that treating obesity reduces medical costs and improves workplace productivity [5]. Despite the strong benefits of GLP‐1 RAs, these medications are not considered cost‐effective by conventional economic models [6], complicating coverage decisions for employer‐sponsored insurance plans.

Insurance decision‐makers often regard obesity medications solely as healthcare expenses and prioritize them for cost‐cutting. However, obesity medications are more accurately understood as integral components of broader workforce strategies that influence employee satisfaction, productivity, and retention [7]. Reductions in coverage for obesity medications may have consequences beyond immediate cost savings: destabilizing the workforce, undermining morale, and increasing turnover among valued employees.

This study assessed how the loss of GLP‐1 RA coverage for obesity treatment affected employee perceptions of their employer. Loss of coverage was hypothesized to lead to negative feelings among employees toward their employers, as well as other adverse work‐related consequences, such as burnout, decreased motivation, and lower workplace satisfaction.

## 2 Methods

This cross‐sectional study analyzed self‐reported survey data from patients who received care at an academic obesity medicine clinic. Eligible participants were adults (aged 18 years or older) insured through large employer‐sponsored health plans that discontinued coverage for GLP‐1 RAs for the treatment of obesity. Participants must have been prescribed a GLP‐1 RA for obesity on or after June 2021 and subsequently lost medication coverage.

Eligible participants were identified through electronic health record (EHR) screening and invited via email to complete a questionnaire administered through Research Electronic Data Capture (REDCap). Enrollment took place between March–May 2024. Participants provided informed consent prior to survey completion. The survey consisted of 50–70 questions, depending on how respondents answered, with response options including Yes/No, Likert scales, check‐all‐that‐apply, or free‐response, and was designed by the study team, primarily consisting of non‐validated instruments. The survey collected demographic information (age, sex, race/ethnicity, income, BMI), employment type, medication use, and experiences following GLP‐1 RA coverage loss. Additional details are provided in the survey instrument included in the (Supporting Information S1: Figure S1).

The primary exposure was loss of employer‐sponsored GLP‐1 RA coverage. The primary outcome was a self‐reported negative impact on the employee's relationship with their employer following GLP‐1 RA coverage loss. Secondary outcomes included considering new employment, feeling devalued by the employer, and experiencing burnout. Independent variables included demographic and socioeconomic factors, specifically race/ethnicity and household income.

Descriptive statistics summarized participant characteristics. Associations between categorical variables were assessed using chi‐square and Fisher's exact tests. Multivariable logistic regression models estimated adjusted odds ratios (aOR) and 95% confidence intervals (CI) for reporting a negative impact on employer relationships based on race/ethnicity and income. All regression models were adjusted for gender, age, BMI, and job category. Statistical analyses were conducted using Stata v.18 software (StataCorp, College Station, TX), and a *p*‐value < 0.05 indicated statistical significance.

Institutional Review Board (IRB) approval was obtained from The University of Texas Health Science Center at Houston and The University of Texas Southwestern Medical Center (IRB # HSC‐SPH‐23‐0854). Confidentiality was maintained by limiting identifiable information in the analytical dataset.

## 3 Results

A total of 847 invitations were sent, and 419 individuals consented (49.47% response rate). Of these, 247 employees completed the full survey and were included in the analytic sample (mean age, 49.27 years; SD, 9.89; mean BMI, 32.85 kg/m^2^; SD, 8.49; 90.28% women). Most participants were non‐Hispanic White (40.08%), followed by non‐Hispanic Black (30.36%), Hispanic (19.84%), and other races/ethnicities (9.72%) (Table 1).

**TABLE 1:** Characteristics of surveyed employees stratified by perceived impact of GLP‐1 RA obesity medication coverage loss on employer relationship (*N* = 247, 2024).

	Negative impact (*n* = 199, 80.57%)	No impact (*n* = 48, 19.43%)	*p*‐value
Age, mean (SD), yrs	49.47 (9.64)	51.01 (10.81)	0.33
BMI, mean (SD), kg/m^2^	32.43 (7.88)	34.62 (10.58)	0.11
Sex, *n* (%)			0.85
Female	180 (80.72)	43 (19.28)	
Male	19 (79.17)	5 (20.83)	
Race and Ethnicity, *n* (%)			**0.004**
Non‐Hispanic White	88 (88.89)	11 (11.11)	
Non‐Hispanic Black	52 (69.33)	23 (30.67)	
Hispanic	37 (75.51)	12 (24.49)	
Other	22 (91.67)	2 (8.33)	
Household income, *n* (%)			**0.015**
< $150,000	125 (76.22)	39 (23.78)	
≥ $150,000	74 (89.16)	9 (10.84)	

*Note:* Significant differences in race/ethnicity and household income were observed between employees reporting a negative impact on employer perception following GLP‐1 RA coverage loss and those reporting no impact. *p* < 0.05 are bold. Definition: Negative impact was defined as participants reporting worsened perceptions of their employer following loss of GLP‐1 RA obesity medication coverage.

Abbreviation: BMI, Body Mass Index; GLP‐1 RA, glucagon‐like peptide‐1 receptor agonist.

Overall, 80.57% reported that losing GLP‐1 RA coverage had a negative impact on their relationship with their employer, while 19.43% reported no change (Table 1). Participants reporting negative impacts did not differ significantly by age, BMI, or gender; however, significant differences between groups were noted by race/ethnicity (*p* = 0.004) and household income (*p* = 0.015).

**FIGURE 1:**
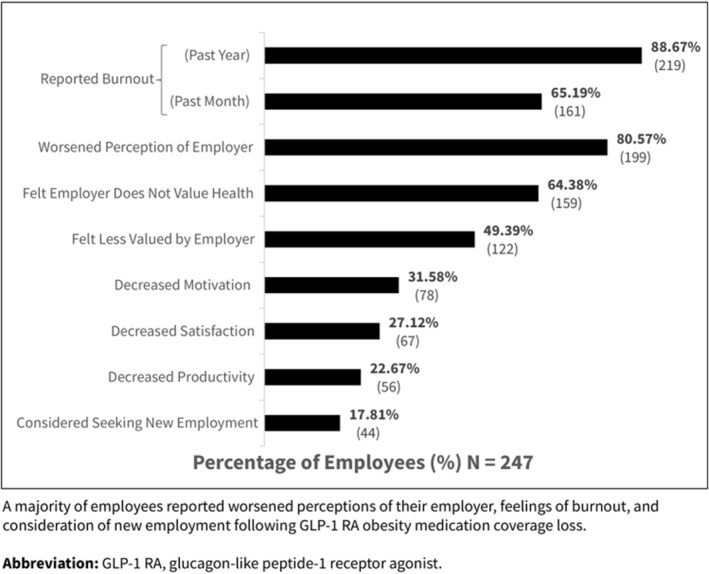
Reported work‐related impacts following GLP‐1 RA coverage loss. A majority of employees reported worsened perceptions of their employer, feelings of burnout, and consideration of new employment following GLP‐1 RA obesity medication coverage loss. GLP‐1 RA, glucagon‐like peptide‐1 receptor agonist.

Beyond disappointment, 17.81% of employees indicated they were considering changing employers due to the loss of coverage. Additionally, 49.39% felt less valued, and 64.38% believed their employer did not value their health. Most participants (88.67%) reported feelings of burnout within the past year, with 65.19% experiencing burnout within the previous month. Many also reported decreased productivity (22.67%), motivation (31.58%), and job satisfaction (27.12%) since losing GLP‐1 RA coverage (Figure 1).

In adjusted logistic regression models (Table 2), non‐Hispanic Black (aOR = 0.29; 95% CI, 0.12–0.70; *p* = 0.006) and Hispanic employees (aOR = 0.34; 95% CI, 0.12–0.93; *p* = 0.036) were significantly less likely than non‐Hispanic White employees to report negative perceptions of their employer. Employees with household incomes ≥$150,000 were more than twice as likely to report negative perceptions than those earning less (aOR = 2.57; 95% CI, 1.14–5.78; *p* = 0.022).

**TABLE 2:** Adjusted odds of reporting negative employer perceptions following GLP‐1 RA obesity medication coverage loss, stratified by race/ethnicity and household income.

Model 1: Odds of negative employer relations after coverage loss by race/ethnicity, gender, age, BMI, and profession			
Race and ethnicity	Odds ratio	95% CI	*p*‐value
Non‐Hispanic Black (NHB)	0.29	(0.12–0.70)	**0.006**
Hispanic	0.34	(0.12–0.93)	**0.036**
Other	1.20	(0.24–5.93)	0.82
Non‐Hispanic White (NHW)	1.00 (ref)	—	—

Model 2: Odds of negative employer relations after coverage loss by household income, adjusted for gender, age, BMI, and profession			
Household income	Odds ratio	95% CI+	*p*‐value
≥ $150,000	2.57	(1.14–5.78)	**0.022**
< $150,000	1.00 (ref)	—	—

*Note:* Employees with higher household incomes were more likely to report worsened perceptions of their employer, while non‐Hispanic Black and Hispanic employees were less likely to report negative employer perceptions compared with non‐Hispanic White employees. *p* < 0.05 are bold. Definition: Negative employer perception was defined as a worsened view of the employer following loss of GLP‐1 RA coverage for obesity treatment.

Abbreviations: aOR, adjusted odds ratio; BMI, Body Mass Index; CI, confidence interval; GLP‐1 RA, glucagon‐like peptide‐1 receptor agonist.

## 4 Discussion

Discontinuation of GLP‐1 RA obesity medication coverage was associated with widespread negative impacts on employee job satisfaction, morale, and burnout. Employees who reported a worsening relationship with their employer after coverage loss made up the majority of those affected, highlighting the consequences for workforce stability. Nearly one in five employees considered leaving their job, underscoring significant risks to retention. Employee turnover is resource‐intensive, costly, and disruptive, especially among highly skilled workers [8]. These costs potentially offset or surpass the intended short‐term savings from reducing benefits.

Employees with a higher household income were more than twice as likely to report worsened employer relationships, suggesting that coverage decisions might lead to turnover among workers in upper‐level positions. This highlights the potential impact on a segment of the workforce that may be particularly challenging and resource‐intensive to replace [9].

Conversely, non‐Hispanic Black and Hispanic employees reported fewer negative perceptions, potentially indicating disparities in perceived job mobility and systemic barriers to employment opportunities [10]. Future research is warranted to explore these disparities and their implications for workforce equity.

Beyond workforce morale and retention, loss of GLP‐1 RA coverage could adversely affect employee health. These medications have been shown to be effective in promoting clinically significant weight loss and improving obesity‐related complications [2, 11]. Obesity and overweight were estimated to cost U.S. employers more than $425 billion per year [5], and weight reductions consistent with GLP‐1 RA clinical trial outcomes could yield over $150 billion in annual healthcare savings [5]. Equity in access to GLP‐1 RA therapies remains a critical concern. Recent evidence suggests that sociodemographic factors, including insurance type, income, and race/ethnicity, significantly influence the initiation of Semaglutide for obesity treatment [12]. Without deliberate efforts to improve equitable access to obesity care, existing disparities in health outcomes and workplace opportunities may widen.

While expanding access to effective obesity medications holds the promise of improving long‐term health outcomes, it also poses substantial financial challenges. A recent economic analysis estimated that expanding Medicare Part D coverage to include GLP‐1 RAs for obesity treatment would improve clinical outcomes but result in a net increase of nearly $48 billion in Medicare spending over 10 years [13].

However, the Centers for Medicare & Medicaid Services (CMS) recently introduced the BALANCE (Better Approaches to Lifestyle and Nutrition for Comprehensive Health) Model, which aims to increase access to GLP‐1 medications for individuals enrolled in Medicare or Medicaid [14]. This model allows CMS to negotiate directly with drug manufacturers on behalf of state Medicaid agencies and Medicare Part D plan sponsors [14]. If private insurers were to adopt similar negotiation strategies, the employer–employee relationships observed in this study could differ under future coverage and pricing conditions.

Given the high and unpredictable costs associated with GLP‐1 RA therapies, employers face substantial uncertainty when designing or maintaining employee health insurance plans. As a result, some employers may interpret these findings as justification to forgo coverage rather than risk offering a benefit that might later be withdrawn due to financial constraints. However, obesity care coverage may be better understood as an investment in workforce stability, productivity, and long‐term organizational health. Additional research is needed to guide employers in making fully informed decisions about major benefit changes.

Limitations included the cross‐sectional design, which restricts causal inference, the absence of a control or comparison group, and the reliance on self‐reported data from non‐validated survey instruments, which may introduce bias. The single‐site sample may limit generalizability, and employer perspectives were not assessed. Future longitudinal studies are needed to assess the temporal relationship between benefit changes and employee outcomes, including turnover, healthcare utilization, and long‐term health effects.

## 5 Conclusion

Removing coverage for GLP‐1 RA obesity medications may erode employee morale, reduce productivity, and increase turnover, with significant organizational and economic consequences. Rather than focusing solely on short‐term savings, employers should consider the long‐term value of investing in comprehensive obesity care, including sustained access to effective medications and bariatric surgery, to support workforce stability and employee well‐being.

## Author Contributions

Jackson Francis had full access to all the data in the study and takes responsibility for the integrity of the data and the accuracy of the data analysis. Concept and Design: Francis, Messiah, Almandoz. Acquisition, analysis, or interpretation of data: All authors. Drafting of the manuscript: Francis, Almandoz. Critical review of the manuscript for important intellectual content: All authors Statistical analysis: Jackson Francis. Supervision: Messiah, Almandoz.

## Funding

The authors have nothing to report.

## Conflicts of Interest

Dr. Almandoz has received advisory or consulting fees from Novo Nordisk, Eli Lilly, Boehringer Ingelheim, AbbVie, Amgen, Kailera, Metsera, Rivus, Rhythm Pharmaceuticals, and Nestlé Health Science.

## Supporting information


Supporting Information S1

## Data Availability

The data supporting this study's findings are not openly available due to reasons of sensitivity and are available from the corresponding author upon reasonable request. Data are in controlled‐access data storage at the UTHealth School of Public Health.
