# Supplementary material for: Negative Consequences of Removing GLP‐1 RA Obesity Coverage: A Cross‐Sectional Cohort Comparison Study
Source: Obes Sci Pract. 2026 Mar 3;12(2):e70123. doi: 10.1002/osp4.70123 (PMC12955695; doi:10.1002/osp4.70123)
Supplement: Supplementary file 1 — Supporting Information S1 [file OSP4-12-e70123-s001.pdf]

Data Dictionary Codebook

GLP-1 Loss of Coverage Study (PID: 35741)

11/21/2025 4:03pm

	#	Variable / Field Name	Field Label<br>*Field Note*	Field Attributes (Field Type, Validation, Choices, Calculations, etc.)
Instrument: **Participant Details** (participant_details)				[collapsed]
Instrument: **Consent** (consent) Enabled as survey				[collapsed]
Instrument: **Demographics** (demographics) Enabled as survey				
	46	[de_start_ts]	Demographics start timestamp	text (datetime_seconds_mdy)<br>Field Annotation: @HIDDEN-PDF @NOW @HIDDEN
	47	[de_start_date]	Demographics start date	text (date_mdy)<br>Field Annotation: @HIDDEN-PDF @TODAY @HIDDEN
	48	[de_header]	Demographics	descriptive
	49	[de_progress_bar]		descriptive<br>Field Annotation: @HIDDEN-PDF
	50	[de_dob]	What is your date of birth?<br>*mm/dd/yyyy*	text (date_mdy, Min: 1900-01-01, Max: today), Required, Identifier<br>Field Annotation: @HIDEBUTTON
	51	[de_age]	Respondent age<br>*years*	calc<br>Calculation: rounddown ((datediff ([de_dob] , [de_start_date] , "y" , "mdy")) , 0)<br>Field Annotation: @HIDDEN-SURVEY
	52	[de_age_cont]	Respondent age, continuous<br>*years*	calc<br>Calculation: rounddown ((datediff ([de_dob] , [de_start_date] , "y" , "mdy")) , 2)<br>Field Annotation: @HIDDEN-SURVEY
	53	[de_mrn_age]	Respondent MRN age<br>*years*	calc<br>Calculation: rounddown ((datediff ([pd_dob] , [de_start_date] , "y" , "mdy")) , 0)<br>Field Annotation: @HIDDEN-SURVEY
	54	[de_mrn_age_cont]	Respondent MRN age, continuous<br>*years*	calc<br>Calculation: rounddown ((datediff ([pd_dob] , [de_start_date] , "y" , "mdy")) , 2)<br>Field Annotation: @HIDDEN-SURVEY
	55	[de_gender]	What is your gender identity?	radio, Required<br>1 | Female<br>2 | Male<br>3 | Non-binary<br>98 | None of these describe me {de_gender_oth}<br>99 | Prefer not to answer
	56	[de_gender_oth]<br><br>Show the field ONLY if:<br>[de_gender] = '98'	Please specify other gender.	text<br>Field Annotation: @PLACEHOLDER = 'Please specify'
	57	[de_race]	What is your race?[Select all that apply.]	checkbox, Required<br>1 | de_race___1 | American Indian or Alaskan Native

				2 | de_race___2 | Asian<br>3 | de_race___3 | Black or African American<br>4 | de_race___4 | Native Hawaiian or Other Pacific Islander<br>5 | de_race___5 | White<br>98 | de_race___98 | Other {de_race_oth}
	58	[de_race_oth]<br>Show the field ONLY if:<br>[de_race(98)] = '1'	Please specify other race.	text<br>Field Annotation: @PLACEHOLDER = 'Please specify'
	59	[de_hispanic]	Are you of Hispanic, Latino/a, or Spanish origin?	yesno, Required<br>1 | Yes<br>0 | No
	60	[de_income]	What is your total annual HOUSEHOLD income?	radio, Required<br>1 | Under $25,000<br>3 | $25,000 to $49,999<br>4 | $50,000 to $74,999<br>5 | $75,000 to $99, 999<br>6 | $100,000 to $149,999<br>7 | $150,000 to $199,999<br>8 | $200,000 and over
	61	[de_beneficiary]	What is your association with the UT System insurance benefits plan?	radio, Required<br>1 | I'm a current employee of the UT System<br>2 | I'm a retired employee of the UT System<br>3 | I'm a spouse/dependent of a UT System employee/retiree
	62	[de_job]<br>Show the field ONLY if:<br>[de_beneficiary] = '1' OR [de_beneficiary] = '2'	What is or was your job description in the UT System?	radio, Required<br>1 | Healthcare clinician {de_job_clinician}<br>2 | Healthcare staff {de_job_staff}<br>3 | Research or teaching faculty<br>4 | Other research<br>5 | Administration, Accounting, or Human Resources<br>6 | Facilities Management<br>98 | Other {de_job_oth}
	63	[de_job_clinician]<br>Show the field ONLY if:<br>[de_job] = '1'	Specify healthcare clinician job description.	radio, Required<br>1 | Physician<br>2 | Dentist<br>3 | Pharmacist<br>4 | Advance Practice Provider<br>5 | Psychologist<br>6 | Allied Health (e.g. RD, PT, OT, SW, LPC)

				98 Other
	64	[de_job_staff]<br>Show the field ONLY if:<br>[de_job] = '2'	Specify healthcare staff job description.	radio, Required<br>1 Manager or Supervisor<br>2 Nurse<br>3 Medical Assistant/Technician<br>4 Patient Care Technician<br>5 Clinical Staff Assistant<br>6 Guest Services<br>98 Other
	65	[de_job_oth]<br>Show the field ONLY if:<br>[de_job] = '98'	Please specify other job.	text<br>Field Annotation: @PLACEHOLDER = 'Please specify'
	66	[demographics_complete]	Section Header: *Form Status*<br>Complete?	dropdown<br>0 Incomplete<br>1 Unverified<br>2 Complete
Instrument: **Weight Loss Medication History** (weight_loss_medication_history) Enabled as survey				
	67	[wmh_start_ts]	Weight loss medication history start timestamp	text (datetime_seconds_mdy)<br>Field Annotation: @HIDDEN-PDF @NOW @HIDDEN
	68	[wmh_start_date]	Weight loss medication history start date	text (date_mdy)<br>Field Annotation: @HIDDEN-PDF @TODAY @HIDDEN
	69	[wmh_header]	Weight Loss Medication History	descriptive
	70	[wmh_progress_bar]	20% Complete	descriptive<br>Field Annotation: @HIDDEN-PDF
	71	[wmh_meds]	Regarding your weight loss medications... Taken in the prior 12 months... Currently taking... {wmh_meds_12m} {wmh_meds_current}	descriptive
	72	[wmh_meds_12m]	Which mediations for weight loss have you taken in the prior 12 MONTHS?[Select all that apply.]	checkbox, Required<br>0 wmh_meds_12m___0 I have not taken a weight loss medica[illegible] in the past 12 mon[illegible]<br>1 wmh_meds_12m___1 Liraglutide (Saxenda/Victoza)<br>2 wmh_meds_12m___2 Semaglutide (Ozempic/Wegovy)<br>3 wmh_meds_12m___3 Tirzepatide (Mounjaro/Zepbo[illegible]<br>4 wmh_meds_12m___4 Buproprion/naltre[illegible] (Contrave)<br>5 wmh_meds_12m___5 Phentermine/topi[illegible] (Qsymia)<br>6 wmh_meds_12m___6 Phentermine (Adipex/Lomira)<br>7 wmh_meds_12m___7 Orlistat (Xenical/Al[illegible]

				8 wmh_meds_12m___8 Topiramate (Topan<br>9 wmh_meds_12m___9 Bupropion (Wellbu<br>10 wmh_meds_12m___10 Metformin (Glucop<br>11 wmh_meds_12m___11 Lisdexamfetamine (Vyvanse)<br>12 wmh_meds_12m___12 Methylphenidate (Concerta)<br>13 wmh_meds_12m___13 Exenatide (Byetta/Bydureon)<br>14 wmh_meds_12m___14 Dulaglutide (Trulici<br>98 wmh_meds_12m___98 Other {wmh_meds_12m_<br>99 wmh_meds_12m___99 I don't know<br><br>Field Annotation: @NONEOFTHEABOVE = '0,99'
	73	[wmh_meds_12m_oth]<br><br>Show the field ONLY if: [wmh_meds_12m(98)] = '1'	Please specify other medications for weight loss in the prior 12 months.	text<br>Field Annotation: @PLACEHOLDER = 'Please specify'
	74	[wmh_meds_current]	Which medications for weight loss are you CURRENTLY taking?[Select all that apply.]	checkbox, Required<br>0 wmh_meds_current___0 I am not curren a medication fo loss<br>1 wmh_meds_current___1 Liraglutide (Saxenda/Victoz<br>2 wmh_meds_current___2 Semaglutide (Ozempic/Wego<br>3 wmh_meds_current___3 Tirzepatide (Mounjaro/Zepb<br>4 wmh_meds_current___4 Buproprion/nal (Contrave)<br>5 wmh_meds_current___5 Phentermine/to (Qsymia)<br>6 wmh_meds_current___6 Phentermine (Adipex/Lomira<br>7 wmh_meds_current___7 Orlistat (Xenica<br>8 wmh_meds_current___8 Topiramate (Top<br>9 wmh_meds_current___9 Bupropion (Wel<br>10 wmh_meds_current___10 Metformin (Glu<br>11 wmh_meds_current___11 Lisdexamfetam (Vyvanse)<br>12 wmh_meds_current___12 Methylphenidat (Concerta)<br>13 wmh_meds_current___13 Exenatide (Byetta/Bydure<br>14 wmh_meds_current___14 Dulaglutide (Tru

				98 | wmh_meds_current___98 | Other {wmh_meds_cu<br>99 | wmh_meds_current___99 | I don't know<br><br>Field Annotation: @NONEOFTHEABOVE = '0,99'
	75	[wmh_meds_current_oth]<br><br>Show the field ONLY if: [wmh_meds_current(98)] = '1'	Please specify other medications for weight loss currently taking.	text<br>Field Annotation: @PLACEHOLDER = 'Please specify'
	76	[weight_loss_medication_history_complete]	Section Header: *Form Status*<br><br>Complete?	dropdown<br>0 | Incomplete<br>1 | Unverified<br>2 | Complete
**Instrument: Medical History (medical_history) Enabled as survey**				
	77	[mh_start_ts]	Medical history start timestamp	text (datetime_seconds_mdy)<br>Field Annotation: @HIDDEN-PDF @NOW @HIDDEN
	78	[mh_start_date]	Medical history start date	text (date_mdy)<br>Field Annotation: @HIDDEN-PDF @TODAY @HIDDEN
	79	[mh_header]	Medical History	descriptive
	80	[mh_progress_bar]	30% Complete	descriptive<br>Field Annotation: @HIDDEN-PDF
	81	[mh_ht]	Section Header: *Please tell us a bit about your weight and health history.*<br><br>What is your height? Feet{mh_ht_ft}ft Inches{mh_ht_in}in * must provide value	descriptive
	82	[mh_ht_ft]	What is your height? (feet only)<br>*ft*	dropdown, Required<br>4 | 4<br>5 | 5<br>6 | 6<br>7 | 7
	83	[mh_ht_in]	What is your height? (inches only)<br>*in*	dropdown, Required<br>0 | 0<br>1 | 1<br>2 | 2<br>3 | 3<br>4 | 4<br>5 | 5<br>6 | 6<br>7 | 7<br>8 | 8<br>9 | 9<br>10 | 10

				11 | 11
	84	[mh_ht_cm]	Height in cm.<br>*cm*	calc<br>Calculation: ([mh_ht_ft] * 30.48) + ([mh_ht_in] * 2.54)<br>Field Annotation: @HIDDEN-PDF @HIDDEN
	85	[mh_wt]	What were your weight... At age 18{mh_wt_18_lbs}lbs Currently{mh_wt_current_lbs}lbs * must provide value	descriptive
	86	[mh_wt_18_lbs]	What was your weight at age 18 years?<br>*lbs*	text (integer, Min: 100, Max: 1000), Required
	87	[mh_wt_18_kg]	Age 18 years weight in kg.<br>*kg*	calc<br>Calculation: [mh_wt_18_lbs] * 0.45359237<br>Field Annotation: @HIDDEN @HIDDEN-PDF
	88	[mh_wt_current_lbs]	What is your CURRENT weight?<br>*lbs*	text (integer, Min: 100, Max: 1000), Required
	89	[mh_wt_current_kg]	Current weight in kg.<br>*kg*	calc<br>Calculation: [mh_wt_current_lbs] * 0.45359237<br>Field Annotation: @HIDDEN @HIDDEN-PDF
	90	[mh_bmi_current_calc]	Current calculated BMI	calc<br>Calculation: ([mh_wt_current_lbs] / ((([mh_ht_ft] * 12) + [mh_ht_in]) * (([mh_ht_ft] * 12) + [mh_ht_in]))) * 703<br>Field Annotation: @HIDDEN
	91	[mh_wt_high]	What has been your heaviest weight as an adult? Heaviest wt{mh_wt_high_lbs}lbs At what age did you reach this weight? Age heaviest wt{mh_wt_high_age_yrs}years * must provide value	descriptive
	92	[mh_wt_high_lbs]	What has been your heaviest weight as an adult?<br>*lbs*	text (integer, Min: 100, Max: 1000), Required
	93	[mh_wt_high_kg]	Heaviest adult weight in kg.<br>*kg*	calc<br>Calculation: [mh_wt_high_lbs] * 0.45359237<br>Field Annotation: @HIDDEN @HIDDEN-PDF
	94	[mh_wt_high_age_yrs]	At what age did you reach this weight?<br>*years*	text (integer, Min: 10, Max: 100), Required
	95	[mh_wt_goal_lbs]	What is your goal maintenance weight?<br>*lbs*	text (integer, Min: 100, Max: 1000), Required
	96	[mh_wt_goal_kg]	Goal maintenance weight in kg.<br>*kg*	calc<br>Calculation: [mh_wt_goal_lbs] * 0.45359237<br>Field Annotation: @HIDDEN @HIDDEN-PDF
	97	[mh_wt_comp]	Do you have a history of any of the following complications related to obesity?[Select all that apply.]	checkbox, Required<br>1 | mh_wt_comp___1 | Type-2 diabetes<br>2 | mh_wt_comp___2 | Pre-diabetes<br>3 | mh_wt_comp___3 | Hypertension<br>4 | mh_wt_comp___4 | Hyperlipidemia<br>5 | mh_wt_comp___5 | Fatty liver<br>6 | mh_wt_comp___6 | Sleep apnea<br>7 | mh_wt_comp___7 | Osteoarthritis<br>0 | mh_wt_comp___0 | None

				Field Annotation: @NONEOFTHEABOVE = '0'
	98	[mh_diabetes_yr]<br><br>Show the field ONLY if:<br>[mh_wt_comp(1)] = '1'	In what year were you diagnosed with diabetes?<br>*year*	text (integer, Min: 1900, Max: 2025)<br>Field Annotation: @PLACEHOLDER = 'XXXX'
	99	[mh_bar_surg]	Have you ever had weight loss surgery?	yesno, Required<br>1 Yes<br>0 No
	100	[mh_bar_surg_proc]<br><br>Show the field ONLY if:<br>[mh_bar_surg] = '1'	What type of surgery did you have?[Select all that apply.]	checkbox, Required<br>1 mh_bar_surg_proc___1 Lap band<br>2 mh_bar_surg_proc___2 Gastric sleeve<br>3 mh_bar_surg_proc___3 Gastric bypass<br>4 mh_bar_surg_proc___4 Biliopancreatic diversion with du switch (BPD-DS)<br>5 mh_bar_surg_proc___5 Endoscopic sleev gastroplasty<br>98 mh_bar_surg_proc___98 Other {mh_bar_surg_pr<br>99 mh_bar_surg_proc___99 I don't know<br><br>Field Annotation: @NONEOFTHEABOVE='99'
	101	[mh_bar_surg_proc_oth]<br><br>Show the field ONLY if:<br>[mh_bar_surg] = '1' AND [mh_bar_surg_proc(98)] = '1'	Please specify other surgery procedure.	text<br>Field Annotation: @PLACEHOLDER = 'Please specify'
	102	[mh_bar_surg_date]<br><br>Show the field ONLY if:<br>[mh_bar_surg] = '1'	When did you have your surgery?[If you had more than one, provide the date of your last bariatric surgery.]<br>*mm/dd/yyyy*	text (date_mdy)<br>Field Annotation: @HIDEBUTTON
	103	[medical_history_complete]	Section Header: *Form Status*<br>Complete?	dropdown<br>0 Incomplete<br>1 Unverified<br>2 Complete
**Instrument: Everyday Discrimination Scale (everyday_discrimination_scale)** **Enabled as survey**				
	104	[eds_start_ts]	Everyday discrimination scale start timestamp	text (datetime_seconds_mdy)<br>Field Annotation: @HIDDEN-PDF @NOW @HIDDEN
	105	[eds_start_date]	Everyday discrimination scale start date	text (date_mdy)<br>Field Annotation: @HIDDEN-PDF @TODAY @HIDDEN
	106	[eds_header]	Everyday Discrimination Scale	descriptive
	107	[eds_progress_bar]	35% Complete	descriptive

	108	[eds_disc_less_courtesy]	Section Header: *Prior being on GLP-1 therapy - in your day-to-day life, how often didany of the following things happen to you because of your weight?*<br>You are treated with less courtesy than other people are.	radio (Matrix), Required<br>0 Never<br>1 Less than once a year<br>2 A few times a year<br>3 A few times a month<br>4 At least once a week<br>5 Almost everyday
	109	[eds_disc_less_respect]	You are treated with less respect than other people are.	radio (Matrix), Required<br>0 Never<br>1 Less than once a year<br>2 A few times a year<br>3 A few times a month<br>4 At least once a week<br>5 Almost everyday
	110	[eds_disc_poor_service]	You receive poorer service than other people at restaurants or stores.	radio (Matrix), Required<br>0 Never<br>1 Less than once a year<br>2 A few times a year<br>3 A few times a month<br>4 At least once a week<br>5 Almost everyday
	111	[eds_disc_not_smart]	People act as if they think you are not smart.	radio (Matrix), Required<br>0 Never<br>1 Less than once a year<br>2 A few times a year<br>3 A few times a month<br>4 At least once a week<br>5 Almost everyday
	112	[eds_disc_act_afraid]	People act as if they are afraid of you.	radio (Matrix), Required<br>0 Never<br>1 Less than once a year<br>2 A few times a year<br>3 A few times a month<br>4 At least once a week<br>5 Almost everyday
	113	[eds_disc_act_dishonest]	People act as if they think you are dishonest.	radio (Matrix), Required<br>0 Never<br>1 Less than once a year<br>2 A few times a year<br>3 A few times a month<br>4 At least once a week

			5 Almost everyday
114	[eds_disc_act_better]	People act as if they're better than you are.	radio (Matrix), Required<br>0 Never<br>1 Less than once a year<br>2 A few times a year<br>3 A few times a month<br>4 At least once a week<br>5 Almost everyday
115	[eds_disc_call_names]	You are called names or insulted.	radio (Matrix), Required<br>0 Never<br>1 Less than once a year<br>2 A few times a year<br>3 A few times a month<br>4 At least once a week<br>5 Almost everyday
116	[eds_disc_threat_harass]	You are threatened or harassed.	radio (Matrix), Required<br>0 Never<br>1 Less than once a year<br>2 A few times a year<br>3 A few times a month<br>4 At least once a week<br>5 Almost everyday
117	[eds_reason_discrim_txt]	What do you think is the MAIN REASON for these experiences?	notes
118	[eds_reason_discrim]<br>Show the field ONLY if: [eds_disc_less_courtesy] = '2' OR [eds_disc_less_courtesy] = '3' OR [eds_disc_less_courtesy] = '4' OR [eds_disc_less_courtesy] = '5' OR [eds_disc_less_respect] = '2' OR [eds_disc_less_respect] = '3' OR [eds_disc_less_respect] = '4' OR [eds_disc_less_respect] = '5' OR [eds_disc_poor_service] = '2' OR [eds_disc_poor_service] = '3' OR [eds_disc_poor_service] = '4' OR [eds_disc_poor_service] = '5' OR [eds_disc_not_smart] = '2' OR [eds_disc_not_smart] = '3' OR [eds_disc_not_smart] = '4' OR [eds_disc_not_smart] = '5' OR [eds_disc_act_afraid] = '2' OR [eds_disc_act_afraid] = '3' OR [eds	For questions where you answered "A few times a year" or more frequently, what do you think is the MAIN REASON for these experiences?	radio<br>1 Your ancestry or national origins<br>2 Your gender<br>3 Your race<br>4 Your age<br>5 Your religion<br>6 Your height<br>7 Your weight<br>8 Some other aspect of your physical appearance<br>9 Your sexual orientation<br>10 Your education or income level<br>11 A physical disability<br>12 Your shade of skin color<br>98 Other {eds_reason_discrim_oth}

		_disc_act_afraid] = '4' OR [eds_disc_act_afraid] = '5' OR [eds_disc_act_dishonest] = '2' OR [eds_disc_act_dishonest] = '3' OR [eds_disc_act_dishonest] = '4' OR [eds_disc_act_dishonest] = '5' OR [eds_disc_act_better] = '2' OR [eds_disc_act_better] = '3' OR [eds_disc_act_better] = '4' OR [eds_disc_act_better] = '5' OR [eds_disc_call_names] = '2' OR [eds_disc_call_names] = '3' OR [eds_disc_call_names] = '4' OR [eds_disc_call_names] = '5' OR [eds_disc_threat_harass] = '2' OR [eds_disc_threat_harass] = '3' OR [eds_disc_threat_harass] = '4' OR [eds_disc_threat_harass] = '5'		
	119	[eds_reason_discrim_oth]<br>Show the field ONLY if: [eds_reason_discrim] = '98'	Please specify other main reason for discrimination.	text<br>Field Annotation: @PLACEHOLDER = 'Please specify'
	120	[everyday_discrimination_scale_complete]	Section Header: *Form Status*<br>Complete?	dropdown<br>0 Incomplete<br>1 Unverified<br>2 Complete
**Instrument: GLP Usage (glp_usage) Enabled as survey**				
	121	[gu_start_ts]	GLP usage start timestamp	text (datetime_seconds_mdy)<br>Field Annotation: @HIDDEN-PDF @NOW @HIDDEN
	122	[gu_start_date]	GLP usage start date	text (date_mdy)<br>Field Annotation: @HIDDEN-PDF @TODAY @HIDDEN
	123	[gu_header]	GLP-1 Usage	descriptive
	124	[gu_progress_bar]	40% Complete	descriptive<br>Field Annotation: @HIDDEN-PDF
	125	[gu_use]	Have you used any GLP-1 medications, such as Ozempic, Wegovy, Mounjaro, Zepbound, etc.?	yesno, Required<br>1 Yes<br>0 No
	126	[gu_qol_improve]<br>Show the field ONLY if: [gu_use] = '1'	Section Header: *How did being on GLP-1 therapy impacted your life?*<br>GLP-1 therapy improved my quality of life.	radio (Matrix), Required<br>1 Strongly disagree<br>2 Disagree<br>3 Neutral<br>4 Agree<br>5 Strongly agree

	127	[gu_qol_control]<br>Show the field ONLY if:<br>[gu_use] = '1'	For the first time, I felt in control of my weight and health.	radio (Matrix), Required<br>1 Strongly disagree<br>2 Disagree<br>3 Neutral<br>4 Agree<br>5 Strongly agree
	128	[gu_imp_confident]<br>Show the field ONLY if:<br>[gu_use] = '1'	Section Header: *As my weight and health improved while taking GLP-1 therapy...*<br>I felt more confident.	radio (Matrix), Required<br>1 Strongly disagree<br>2 Disagree<br>3 Neutral<br>4 Agree<br>5 Strongly agree
	129	[gu_imp_serious]<br>Show the field ONLY if:<br>[gu_use] = '1'	People took me more seriously.	radio (Matrix), Required<br>1 Strongly disagree<br>2 Disagree<br>3 Neutral<br>4 Agree<br>5 Strongly agree
	130	[gu_imp_work]<br>Show the field ONLY if:<br>[gu_use] = '1'	People started looking past my body size and saw my worth.	radio (Matrix), Required<br>1 Strongly disagree<br>2 Disagree<br>3 Neutral<br>4 Agree<br>5 Strongly agree
	131	[gu_imp_social]<br>Show the field ONLY if:<br>[gu_use] = '1'	I felt less distress in social interactions.	radio (Matrix), Required<br>1 Strongly disagree<br>2 Disagree<br>3 Neutral<br>4 Agree<br>5 Strongly agree
	132	[gu_imp_relationship]<br>Show the field ONLY if:<br>[gu_use] = '1'	I felt less distress in relationship interactions (e.g., with partner or in dating).	radio (Matrix), Required<br>1 Strongly disagree<br>2 Disagree<br>3 Neutral<br>4 Agree<br>5 Strongly agree
	133	[gu_imp_professional]<br>Show the field ONLY if:<br>[gu_use] = '1'	I felt less distress in professional interactions.	radio (Matrix), Required<br>1 Strongly disagree<br>2 Disagree<br>3 Neutral<br>4 Agree

			5 Strongly agree
134	[gu_imp_career]<br>Show the field ONLY if:<br>[gu_use] = '1'	I was able to advance in my career with fewer barriers.	radio (Matrix), Required<br>1 Strongly disagree<br>2 Disagree<br>3 Neutral<br>4 Agree<br>5 Strongly agree
135	[glp_usage_complete]	Section Header: *Form Status*<br>Complete?	dropdown<br>0 Incomplete<br>1 Unverified<br>2 Complete

Instrument: GLP Semaglutide History (glp_semaglutide_history) Enabled as survey

136	[gsh_start_ts]	GLP semaglutide history start timestamp	text (datetime_seconds_mdy)<br>Field Annotation: @HIDDEN-PDF @NOW @HIDDEN
137	[gsh_start_date]	GLP semaglutide history start date	text (date_mdy)<br>Field Annotation: @HIDDEN-PDF @TODAY @HIDDEN
138	[gsh_header]	Semaglutide History	descriptive
139	[gsh_progress_bar]	45% Complete	descriptive<br>Field Annotation: @HIDDEN-PDF
140	[gsh_sema_start_date]	Section Header: *Regarding your use of semaglutide (e.g., Ozempic or Wegovy)...*<br>When did you start taking semaglutide?[Provide an approximate date if unsure of exactly when you started.]<br>*mm/dd/yyyy*	text (date_mdy, Min: [pd_dob], Max: today), Required<br>Field Annotation: @HIDEBUTTON
141	[gsh_wt_sema]	What was your weight... Started semaglutide{gsh_wt_sema_start_lbs}lbs Lowest wt w/ semaglutide{gsh_wt_sema_low_lbs}lbs	descriptive
142	[gsh_wt_sema_start_lbs]	What was your weight when you started taking semaglutide?<br>*lbs*	text (integer, Min: 100, Max: 1000), Required
143	[gsh_wt_sema_start_kg]	Start semaglutide weight in kg.<br>*kg*	calc<br>Calculation: [gsh_wt_sema_start_lbs] * 0.45359237<br>Field Annotation: @HIDDEN @HIDDEN-PDF
144	[gsh_wt_sema_low_lbs]	What was your lowest weight while taking semaglutide?<br>*lbs*	text (integer, Min: 100, Max: 1000), Required
145	[gsh_wt_sema_low_kg]	Semaglutide lowest weight in kg.<br>*kg*	calc<br>Calculation: [gsh_wt_sema_low_lbs] * 0.45359237<br>Field Annotation: @HIDDEN @HIDDEN-PDF
146	[gsh_sema_last_date]	When was your last dose of semaglutide? [Provide an approximate date if unsure of exactly when you had your last dose.]<br>*mm/dd/yyyy*	text (date_mdy, Min: 2017-01-01, Max: today), Required
147	[gsh_subheader]	To continue taking semaglutide...	descriptive

		Show the field ONLY if: [wmh_meds_current(2)] = '1'		
	148	[gsh_take_stock]	I stockpiled a supply of medication.	radio (Matrix), Required<br>1 Yes<br>0 No
	149	[gsh_take_pay_usa]	I cash-paid from a source in the USA.	radio (Matrix), Required<br>1 Yes<br>0 No
	150	[gsh_take_pay_foreign]	I cash-paid from a source outside the USA.	radio (Matrix), Required<br>1 Yes<br>0 No
	151	[gsh_take_pay_comp]	I purchased compounded medication.	radio (Matrix), Required<br>1 Yes<br>0 No
	152	[gsh_take_consider_comp]	I considered purchasing compounded medication.	radio (Matrix), Required<br>1 Yes<br>0 No
	153	[gsh_take_fda_comp]	Are you aware the Food and Drug Administration advises against the sale and use of compounded semaglutide?	radio (Matrix), Required<br>1 Yes<br>0 No
	154	[gsh_sema_cost_mth]<br>Show the field ONLY if: [gsh_take_pay_usa] = '1' OR [gsh_take_pay_foreign] = '1' OR [gsh_take_pay_comp] = '1'	What is your MONTHLY out-of-pocket cost for semaglutide?<br>*dollars*	slider (number, Min: 0, Max: 2000), Required<br>Slider labels: $0, , $2000<br>Custom alignment: RH
	155	[gsh_sema_diff_find]	It is difficult to find semaglutide.	radio (Matrix), Required<br>1 Strongly disagree<br>2 Disagree<br>3 Neutral<br>4 Agree<br>5 Strongly agree
	156	[gsh_sema_last]	How long after September 1, 2023 did or will your supply last?	radio (Matrix), Required<br>1 0-1 month<br>2 1-3 months<br>3 3-6 months<br>4 6-12 months
	157	[gsh_sema_stock_gone]<br>Show the field ONLY if: [gsh_take_stock] = '1'	What will you do after you run out of supply? [Select all that apply.]	checkbox, Required<br>1 gsh_sema_stock_gone___1 I plan to reduce my dose so the medication lasts longer

				2 | gsh_sema_stock_gone___2 | I plan to take the medication less frequently to make it last longer<br>3 | gsh_sema_stock_gone___3 | I plan to switch to an alternative weight loss medication<br>4 | gsh_sema_stock_gone___4 | I plan to stop using weight loss medications and focus on lower cost lifestyle changes<br>5 | gsh_sema_stock_gone___5 | I plan to have bariatric (weight loss) surgery<br>6 | gsh_sema_stock_gone___6 | I plan to give up on my weight loss journey<br>98 | gsh_sema_stock_gone___98 | Other<br>99 | gsh_sema_stock_gone___99 | I don't know<br><br>Field Annotation: @NONEOFTHEABOVE = '99'
	158	[glp_semaglutide_history_complete]	Section Header: *Form Status*<br>Complete?	dropdown<br>0 | Incomplete<br>1 | Unverified<br>2 | Complete
Instrument: **GLP Tirzepatide History** (glp_tirzepatide_history) **Enabled as survey**				
	159	[gth_start_ts]	GLP tirzepatide history start timestamp	text (datetime_seconds_mdy)<br>Field Annotation: @HIDDEN-PDF @NOW @HIDDEN
	160	[gth_start_date]	GLP tirzepatide history start date	text (date_mdy)<br>Field Annotation: @HIDDEN-PDF @TODAY @HIDDEN
	161	[gth_header]	Tirzepatide History	descriptive
	162	[gth_progress_bar]	50% Complete	descriptive<br>Field Annotation: @HIDDEN-PDF
	163	[gth_tzp_start_date]	Section Header: *Regarding your use of tirzepatide (e.g., Mounjaro or Zepbound)...*	text (date_mdy, Min: 2022-01-01, Max: today), Required<br>Field Annotation: @HIDEBUTTON

			When did you start taking tirzepatide?[Provide an approximate date if unsure of exactly when you started.]<br>*mm/dd/yyyy*	
	164	[gsh_wt_tzp]	What was your weight... Started tirzepatide{gth_wt_tzp_start_lbs}lbs Lowest wt w/ tirzepatide{gth_wt_tzp_low_lbs}lbs	descriptive
	165	[gth_wt_tzp_start_lbs]	What was your weight when you started taking tirzepatide?<br>*lbs*	text (integer, Min: 100, Max: 1000), Required
	166	[gth_wt_tzp_start_kg]	Start tirzepatide weight in kg.<br>*kg*	calc<br>Calculation: [gth_wt_tzp_start_lbs] * 0.45359237<br>Field Annotation: @HIDDEN @HIDDEN-PDF
	167	[gth_wt_tzp_low_lbs]	What was your lowest weight while taking tirzepatide?<br>*lbs*	text (integer, Min: 100, Max: 1000), Required
	168	[gth_wt_tzp_low_kg]	Tirzepatide lowest weight in kg.<br>*kg*	calc<br>Calculation: [gth_wt_tzp_low_lbs] * 0.45359237<br>Field Annotation: @HIDDEN @HIDDEN-PDF
	169	[gth_tzp_last_date]	When was your last dose of tirzepatide?[Provide an approximate date if unsure of exactly when you had your last dose.]<br>*mm/dd/yyyy*	text (date_mdy, Min: 2022-01-01, Max: today), Required
	170	[gth_subheader]<br>Show the field ONLY if: [wmh_meds_current(3)] = '1'	To continue taking tirzepatide...	descriptive
	171	[gth_take_stock]	I stockpiled a supply of medication.	radio (Matrix), Required<br>1 Yes<br>0 No
	172	[gth_take_pay_usa]	I cash-paid from a source in the USA.	radio (Matrix), Required<br>1 Yes<br>0 No
	173	[gth_take_pay_foreign]	I cash-paid from a source outside the USA.	radio (Matrix), Required<br>1 Yes<br>0 No
	174	[gth_take_pay_comp]	I purchased compounded medication.	radio (Matrix), Required<br>1 Yes<br>0 No
	175	[gth_take_consider_comp]	I considered purchasing compounded medication.	radio (Matrix), Required<br>1 Yes<br>0 No
	176	[gth_take_fda_comp]	Are you aware the Food and Drug Administration advises against the sale and use of compounded tirzepatide?	radio (Matrix), Required<br>1 Yes<br>0 No
	177	[gth_tzp_cost_mth]	What is your MONTHLY out-of-pocket cost for tirzepatide?	slider (number, Min: 0, Max: 2000), Required<br>Slider labels: $0, , $2000

		Show the field ONLY if:<br>[gth_take_pay_usa] = '1' OR [gth_take_pay_foreign] = '1' OR [gth_take_pay_comp] = '1'	*dollars*	Custom alignment: RH
	178	[gth_tzp_diff_find]	It is difficult to find tirzepatide.	radio (Matrix), Required<br>1 Strongly disagree<br>2 Disagree<br>3 Neutral<br>4 Agree<br>5 Strongly agree
	179	[gth_tzp_last]	How long after September 1, 2023 did or will your supply last?	radio (Matrix), Required<br>1 0-1 month<br>2 1-3 months<br>3 3-6 months<br>4 6-12 months
	180	[gth_tzp_stock_gone]<br>Show the field ONLY if:<br>[gth_take_stock] = '1'	What will you do after you run out of supply? [Select all that apply.]	checkbox, Required<br>1 gth_tzp_stock_gone___1 I plan to reduce my dose so the medication lasts longer<br>2 gth_tzp_stock_gone___2 I plan to take the medication less frequently to make it last longer<br>3 gth_tzp_stock_gone___3 I plan to switch to an alternative weight loss medication<br>4 gth_tzp_stock_gone___4 I plan to stop using weight loss medications and focus on lower cost lifestyle changes<br>5 gth_tzp_stock_gone___5 I plan to have bariatric (weight loss) surgery<br>6 gth_tzp_stock_gone___6 I plan to give up on my weight loss journey<br>98 gth_tzp_stock_gone___98 Other

			99 | gth_tzp_stock_gone___99 | I don't know<br><br>Field Annotation: @NONEOFTHEABOVE = '99'
181	[glp_tirzepatide_history_complete]	Section Header: *Form Status*<br>Complete?	dropdown<br>0 | Incomplete<br>1 | Unverified<br>2 | Complete
**Instrument: GLP Weight Loss Attempts (glp_weight_loss_attempts)** **Enabled as survey**			
182	[gwa_start_ts]	GLP weight loss attempts start timestamp	text (datetime_seconds_mdy)<br>Field Annotation: @HIDDEN-PDF @NOW @HIDDEN
183	[gwa_start_date]	GLP weight loss attempts start date	text (date_mdy)<br>Field Annotation: @HIDDEN-PDF @TODAY @HIDDEN
184	[gwa_header]	GLP Weight Loss Attempts	descriptive
185	[gwa_progress_bar]	55% Complete	descriptive
186	[gwa_consult_hcp]	Section Header: *Prior to enrolling in the UT Southwestern Weight Wellness Program...*<br>Which of the following had you consulted for weight loss?[Select all that apply.]	checkbox, Required<br>1 | gwa_consult_hcp___1 | Primary care provider<br>2 | gwa_consult_hcp___2 | Obesity medicine specialist<br>3 | gwa_consult_hcp___3 | Other medicine specialist (e.g. cardiologist)<br>4 | gwa_consult_hcp___4 | Bariatric surgeon<br>5 | gwa_consult_hcp___5 | Dietitian<br>6 | gwa_consult_hcp___6 | Exercise specialist/Personal trainer<br>98 | gwa_consult_hcp___98 | Other<br>0 | gwa_consult_hcp___0 | None<br><br>Field Annotation: @NONEOFTHEABOVE = '0'
187	[gwa_tried]	Section Header: *Prior to taking any GLP-1 therapy, which of the following had you tried?*<br>* must provide value Diets Phone Applications "Apps" Commercial Weight Loss Programs Online Weight Loss Platforms {gwa_tried_diet} {gwa_tried_app} {gwa_tried_commercial} {gwa_tried_online} Home Delivery Meal Prep Kits (e.g. Hello Fresh, Blue Apron) Prepared Meals (e.g. Factor, Snap Kitchen, Jenny Craig) Online counseling or coaching (e.g. Berry Street, Nourish, Atwell) {gwa_tried_kit} {gwa_tried_meal} {gwa_tried_coach}	descriptive
188	[gwa_tried_diet]	Prior to taking any GLP-1 therapy, which of the following had you tried: Diets[Select all that apply.]	checkbox, Required<br>0 | gwa_tried_diet___0 | None<br>1 | gwa_tried_diet___1 | Low calorie<br>2 | gwa_tried_diet___2 | Low fat

				3 gwa_tried_diet___3 Low carb<br>4 gwa_tried_diet___4 Keto<br>5 gwa_tried_diet___5 Vegetarian/Vegan<br>6 gwa_tried_diet___6 Mediterranean<br>7 gwa_tried_diet___7 Whole 30<br>8 gwa_tried_diet___8 Paleo<br>9 gwa_tried_diet___9 Intermittent fasting<br>98 gwa_tried_diet___98 Other<br><br>Custom alignment: LV<br>Field Annotation: @NONEOFTHEABOVE = '0'
	189	[gwa_tried_app]	Prior to taking any GLP-1 therapy, which of the following had you tried: Phone Applications "Apps"[Select all that apply.]	checkbox, Required<br>0 gwa_tried_app___0 None<br>1 gwa_tried_app___1 Calorie King<br>2 gwa_tried_app___2 Cronometer<br>3 gwa_tried_app___3 FatSecret<br>4 gwa_tried_app___4 Fooducate<br>5 gwa_tried_app___5 Lose It!<br>6 gwa_tried_app___6 MyFitness Pal<br>7 gwa_tried_app___7 MyNet Diary<br>8 gwa_tried_app___8 Noom<br>9 gwa_tried_app___9 Yazio<br>98 gwa_tried_app___98 Other<br><br>Custom alignment: LV<br>Field Annotation: @NONEOFTHEABOVE = '0'
	190	[gwa_tried_commercial]	Prior to taking any GLP-1 therapy, which of the following had you tried: Commercial Weight Loss Programs[Select all that apply.]	checkbox, Required<br>0 gwa_tried_commercial___0 None<br>1 gwa_tried_commercial___1 Weight Watchers<br>2 gwa_tried_commercial___2 Jenny Craig<br>3 gwa_tried_commercial___3 NutriSystem<br>4 gwa_tried_commercial___4 South Beach<br>5 gwa_tried_commercial___5 Optifast<br>6 gwa_tried_commercial___6 Ideal Protein<br>7 gwa_tried_commercial___7 Huel<br>8 gwa_tried_commercial___8 HMR<br>9 gwa_tried_commercial___9 Slim Fast<br>98 gwa_tried_commercial___98 Other<br><br>Custom alignment: LV<br>Field Annotation: @NONEOFTHEABOVE = '0'

	191	[gwa_tried_kit]	Prior to taking any GLP-1 therapy, have you tried: Home Delivery Meal Prep Kit	yesno, Required<br>1 Yes<br>0 No<br><br>Custom alignment: LV
	192	[gwa_tried_meal]	Prior to taking any GLP-1 therapy, have you tried: Prepared Meals	yesno, Required<br>1 Yes<br>0 No<br><br>Custom alignment: LV
	193	[gwa_tried_online]	Prior to taking any GLP-1 therapy, which of the following had you tried: Online weight loss platform[Select all that apply.]	checkbox, Required<br>0 gwa_tried_online___0 None<br>1 gwa_tried_online___1 Noom<br>2 gwa_tried_online___2 Virta<br>3 gwa_tried_online___3 Calibrate<br>4 gwa_tried_online___4 Roman/Ro<br>5 gwa_tried_online___5 Weight Watchers<br>6 gwa_tried_online___6 Found<br>7 gwa_tried_online___7 Wondr<br>8 gwa_tried_online___8 Naturally Slim<br>9 gwa_tried_online___9 Slim for Life<br>10 gwa_tried_online___10 Omada<br>98 gwa_tried_online___98 Other<br><br>Custom alignment: LV<br>Field Annotation: @NONEOFTHEABOVE = '0'
	194	[gwa_tried_online_rx]<br><br>Show the field ONLY if: [gwa_tried_online(1)] = '1' OR [gwa_tried_online(2)] = '1' OR [gwa_tried_online(3)] = '1' OR [gwa_tried_online(4)] = '1' OR [gwa_tried_online(5)] = '1' OR [gwa_tried_online(6)] = '1' OR [gwa_tried_online(7)] = '1' OR [gwa_tried_online(8)] = '1' OR [gwa_tried_online(9)] = '1' OR [gwa_tried_online(10)] = '1' OR [gwa_tried_online(98)] = '1'	Did you receive a weight loss medication from [gwa_tried_online:checked]?	yesno, Required<br>1 Yes<br>0 No
	195	[gwa_tried_coach]	Prior to taking any GLP-1 therapy, have you tried: Online counseling or coaching for weight loss	yesno, Required<br>1 Yes<br>0 No<br><br>Custom alignment: LV
	196	[gwa_sug]	Section Header: *Which of the following, if any, would you say should be used while taking GLP-1 therapy?*	descriptive

			* must provide value Diets Phone Applications "Apps" Commercial Weight Loss Programs Online Weight Loss Platforms {gwa_sug_diet} {gwa_sug_app} {gwa_sug_commercial} {gwa_sug_online} Home Delivery Meal Prep Kits (e.g. Hello Fresh, Blue Apron) Prepared Meals (e.g. Factor, Snap Kitchen, Jenny Craig) Online counseling or coaching (e.g. Berry Street, Nourish, Atwell) {gwa_sug_kit} {gwa_sug_meal} {gwa_sug_coach}	
	197	[gwa_sug_diet]	Which of the following, if any, would you say should be used while taking GLP-1 therapy: Diets[Select all that apply.]	checkbox, Required<br>0 gwa_sug_diet___0 None<br>1 gwa_sug_diet___1 Low calorie<br>2 gwa_sug_diet___2 Low fat<br>3 gwa_sug_diet___3 Low carb<br>4 gwa_sug_diet___4 Keto<br>5 gwa_sug_diet___5 Vegetarian/Vegan<br>6 gwa_sug_diet___6 Mediterranean<br>7 gwa_sug_diet___7 Whole 30<br>8 gwa_sug_diet___8 Paleo<br>9 gwa_sug_diet___9 Intermittent fasting<br>98 gwa_sug_diet___98 Other<br><br>Custom alignment: LV<br>Field Annotation: @NONEOFTHEABOVE = '0'
	198	[gwa_sug_app]	Which of the following, if any, would you say should be used while taking GLP-1 therapy: Phone Applications "Apps"	checkbox, Required<br>0 gwa_sug_app___0 None<br>1 gwa_sug_app___1 Calorie King<br>2 gwa_sug_app___2 Cronometer<br>3 gwa_sug_app___3 FatSecret<br>4 gwa_sug_app___4 Fooducate<br>5 gwa_sug_app___5 Lose It!<br>6 gwa_sug_app___6 MyFitness Pal<br>7 gwa_sug_app___7 MyNet Diary<br>8 gwa_sug_app___8 Noom<br>9 gwa_sug_app___9 Yazio<br>98 gwa_sug_app___98 Other<br><br>Custom alignment: LV<br>Field Annotation: @NONEOFTHEABOVE = '0'
	199	[gwa_sug_commercial]	Which of the following, if any, would you say should be used while taking GLP-1 therapy: Commercial Weight Loss Programs[Select all that apply.]	checkbox, Required<br>0 gwa_sug_commercial___0 None<br>1 gwa_sug_commercial___1 Weight Watchers<br>2 gwa_sug_commercial___2 Jenny Craig<br>3 gwa_sug_commercial___3 NutriSystem

				4 gwa_sug_commercial___4 South Beach<br>5 gwa_sug_commercial___5 Optifast<br>6 gwa_sug_commercial___6 Ideal Protein<br>7 gwa_sug_commercial___7 Huel<br>8 gwa_sug_commercial___8 HMR<br>9 gwa_sug_commercial___9 Slim Fast<br>98 gwa_sug_commercial___98 Other<br><br>Custom alignment: LV<br>Field Annotation: @NONEOFTHEABOVE = '0'
	200	[gwa_sug_kit]	Should the following be used while taking GLP-1 therapy: Home Delivery Meal Prep Kit	yesno, Required<br>1 Yes<br>0 No<br><br>Custom alignment: LV
	201	[gwa_sug_meal]	Should the following be used while taking GLP-1 therapy: Prepared Meals	yesno, Required<br>1 Yes<br>0 No<br><br>Custom alignment: LV
	202	[gwa_sug_online]	Which of the following, if any, would you say should be used while taking GLP-1 therapy: Online weight loss platform[Select all that apply.]	checkbox, Required<br>0 gwa_sug_online___0 None<br>1 gwa_sug_online___1 Noom<br>2 gwa_sug_online___2 Virta<br>3 gwa_sug_online___3 Calibrate<br>4 gwa_sug_online___4 Roman/Ro<br>5 gwa_sug_online___5 Weight Watchers<br>6 gwa_sug_online___6 Found<br>7 gwa_sug_online___7 Wondr<br>8 gwa_sug_online___8 Naturally Slim<br>9 gwa_sug_online___9 Slim for Life<br>10 gwa_sug_online___10 Omada<br>98 gwa_sug_online___98 Other<br><br>Custom alignment: LV<br>Field Annotation: @NONEOFTHEABOVE = '0'
	203	[gwa_sug_coach]	Should the following be used while taking GLP-1 therapy: Online counseling or coaching for weight loss	yesno, Required<br>1 Yes<br>0 No<br><br>Custom alignment: LV
	204	[gwa_rplc]	Section Header: *In your opinion, which of the following are adequate replacements for GLP-1 therapy?*	descriptive

			* must provide value Diets Phone Applications "Apps" Commercial Weight Loss Programs Online Weight Loss Platforms {gwa_rplc_diet} {gwa_rplc_app} {gwa_rplc_commercial} {gwa_rplc_online} Home Delivery Meal Prep Kits (e.g. Hello Fresh, Blue Apron) Prepared Meals (e.g. Factor, Snap Kitchen, Jenny Craig) Online counseling or coaching (e.g. Berry Street, Nourish, Atwell) {gwa_rplc_kit} {gwa_rplc_meal} {gwa_rplc_coach}	
	205	[gwa_rplc_diet]	In your opinion, which of the following are adequate replacements for GLP-1 therapy: Diets[Select all that apply.]	checkbox, Required<br>0 | gwa_rplc_diet___0 | None<br>1 | gwa_rplc_diet___1 | Low calorie<br>2 | gwa_rplc_diet___2 | Low fat<br>3 | gwa_rplc_diet___3 | Low carb<br>4 | gwa_rplc_diet___4 | Keto<br>5 | gwa_rplc_diet___5 | Vegetarian/Vegan<br>6 | gwa_rplc_diet___6 | Mediterranean<br>7 | gwa_rplc_diet___7 | Whole 30<br>8 | gwa_rplc_diet___8 | Paleo<br>9 | gwa_rplc_diet___9 | Intermittent fasting<br>98 | gwa_rplc_diet___98 | Other<br><br>Custom alignment: LV<br>Field Annotation: @NONEOFTHEABOVE = '0'
	206	[gwa_rplc_app]	In your opinion, which of the following are adequate replacements for GLP-1 therapy: Phone Applications "Apps"[Select all that apply.]	checkbox, Required<br>0 | gwa_rplc_app___0 | None<br>1 | gwa_rplc_app___1 | Calorie King<br>2 | gwa_rplc_app___2 | Cronometer<br>3 | gwa_rplc_app___3 | FatSecret<br>4 | gwa_rplc_app___4 | Fooducate<br>5 | gwa_rplc_app___5 | Lose It!<br>6 | gwa_rplc_app___6 | MyFitness Pal<br>7 | gwa_rplc_app___7 | MyNet Diary<br>8 | gwa_rplc_app___8 | Noom<br>9 | gwa_rplc_app___9 | Yazio<br>98 | gwa_rplc_app___98 | Other<br><br>Custom alignment: LV<br>Field Annotation: @NONEOFTHEABOVE = '0'
	207	[gwa_rplc_commercial]	In your opinion, which of the following are adequate replacements for GLP-1 therapy: Commercial Weight Loss Programs[Select all that apply.]	checkbox, Required<br>0 | gwa_rplc_commercial___0 | None<br>1 | gwa_rplc_commercial___1 | Weight Watchers<br>2 | gwa_rplc_commercial___2 | Jenny Craig<br>3 | gwa_rplc_commercial___3 | NutriSystem

				4 | gwa_rplc_commercial___4 | South Beach<br>5 | gwa_rplc_commercial___5 | Optifast<br>6 | gwa_rplc_commercial___6 | Ideal Protein<br>7 | gwa_rplc_commercial___7 | Huel<br>8 | gwa_rplc_commercial___8 | HMR<br>9 | gwa_rplc_commercial___9 | Slim Fast<br>98 | gwa_rplc_commercial___98 | Other<br><br>Custom alignment: LV<br>Field Annotation: @NONEOFTHEABOVE = '0'
	208	[gwa_rplc_online]	In your opinion, which of the following are adequate replacements for GLP-1 therapy: Online weight loss platform[Select all that apply.]	checkbox, Required<br>0 | gwa_rplc_online___0 | None<br>1 | gwa_rplc_online___1 | Noom<br>2 | gwa_rplc_online___2 | Virta<br>3 | gwa_rplc_online___3 | Calibrate<br>4 | gwa_rplc_online___4 | Roman/Ro<br>5 | gwa_rplc_online___5 | Weight Watchers<br>6 | gwa_rplc_online___6 | Found<br>7 | gwa_rplc_online___7 | Wondr<br>8 | gwa_rplc_online___8 | Naturally Slim<br>9 | gwa_rplc_online___9 | Slim for Life<br>10 | gwa_rplc_online___10 | Omada<br>98 | gwa_rplc_online___98 | Other<br><br>Custom alignment: LV<br>Field Annotation: @NONEOFTHEABOVE = '0'
	209	[gwa_rplc_kit]	Are the following adequate replacements for GLP-1 therapy: Home Delivery Meal Prep Kit	yesno, Required<br>1 | Yes<br>0 | No<br><br>Custom alignment: LV
	210	[gwa_rplc_meal]	Are the following adequate replacements for GLP-1 therapy: Prepared Meals	yesno, Required<br>1 | Yes<br>0 | No<br><br>Custom alignment: LV
	211	[gwa_rplc_coach]	Are the following adequate replacements for GLP-1 therapy: Online counseling or coaching for weight loss	yesno, Required<br>1 | Yes<br>0 | No<br><br>Custom alignment: LV
	212	[glp_weight_loss_attempts_complete]	Section Header: *Form Status*<br>Complete?	dropdown<br>0 | Incomplete

				1 Unverified<br>2 Complete
**Instrument: GLP Weight Loss Attempts Quantified (glp_weight_loss_attempts_quantified)** Enabled as survey				
	213	[gwaq_start_ts]	GLP weight loss attempts quantified start timestamp	text (datetime_seconds_mdy)<br>Field Annotation: @HIDDEN-PDF @NOW @HIDDEN
	214	[gwaq_start_date]	GLP weight loss attempts quantified start date	text (date_mdy)<br>Field Annotation: @HIDDEN-PDF @TODAY @HIDDEN
	215	[gwaq_header]	GLP Weight Loss Attempts Quantified	descriptive
	216	[gwaq_progress_bar]	60% Complete	descriptive
	217	[gwaq_wt_lost_max_lbs]	Section Header: *Prior to taking any GLP-1 therapy...*<br>What is the most weight you have lost?<br>*lbs*	text (integer, Min: 0, Max: 1000), Required
	218	[gwaq_wt_lost_mode]	Which was the most helpful for weight loss?	radio, Required<br>1 Weight loss medication(s)<br>2 Weight loss surgery (bariatric surgery)<br>3 Dieting<br>4 Exercise<br>98 Other<br>0 None<br><br>Custom alignment: LV
	219	[gwaq_wt_lost_20]<br><br>Show the field ONLY if:<br>[gwaq_wt_lost_max_lbs] >= 20 AND ([gwaq_wt_lost_mode] = '1' OR [gwaq_wt_lost_mode] = '2' OR [gwaq_wt_lost_mode] = '3' OR [gwaq_wt_lost_mode] = '4' OR [gwaq_wt_lost_mode] = '98' OR [gwaq_wt_lost_mode] = '0')	Section Header: *Prior to GLP-1 therapy, how many times in your life have you lost...*<br>...20 pounds or more?<br>*times*	text (integer, Min: 0, Max: 99), Required<br>Field Annotation: @PLACEHOLDER = 'Provide an integer value'
	220	[gwaq_wt_lost_35]<br><br>Show the field ONLY if:<br>[gwaq_wt_lost_max_lbs] >= 35 AND ([gwaq_wt_lost_mode] = '1' OR [gwaq_wt_lost_mode] = '2' OR [gwaq_wt_lost_mode] = '3' OR [gwaq_wt_lost_mode] = '4' OR [gwaq_wt_lost_mode] = '98' OR [gwaq_wt_lost_mode] = '0')	...35 pounds or more?<br>*times*	text (integer, Min: 0, Max: 99), Required<br>Field Annotation: @PLACEHOLDER = 'Provide an integer value'
	221	[gwaq_wt_lost_50]<br><br>Show the field ONLY if:<br>[gwaq_wt_lost_max_lbs] >= 50 AND ([gwaq_wt_lost_mode] = '1' OR [gwaq_wt_lost_mode] = '2' OR [g	...50 pounds or more?<br>*times*	text (integer, Min: 0, Max: 99), Required<br>Field Annotation: @PLACEHOLDER = 'Provide an integer value'

		waq_wt_lost_mode] = '3' OR [gwaq_wt_lost_mode] = '4' OR [gwaq_wt_lost_mode] = '98' OR [gwaq_wt_lost_mode] = '0')		
	222	[gwaq_wt_lost_100]<br><br>Show the field ONLY if: [gwaq_wt_lost_max_lbs] >= 100 AND ([gwaq_wt_lost_mode] = '1' OR [gwaq_wt_lost_mode] = '2' OR [gwaq_wt_lost_mode] = '3' OR [gwaq_wt_lost_mode] = '4' OR [gwaq_wt_lost_mode] = '98' OR [gwaq_wt_lost_mode] = '0')	...100 pounds or more?<br>*times*	text (integer, Min: 0, Max: 99), Required<br>Field Annotation: @PLACEHOLDER = 'Provide an integer value'
	223	[glp_weight_loss_attempts_quantified_complete]	Section Header: *Form Status*<br>Complete?	dropdown<br>0 Incomplete<br>1 Unverified<br>2 Complete
**Instrument: GLP Losing Coverage Effects (glp_losing_coverage_effects)**				**Enabled as survey**
	224	[glce_start_ts]	GLP losing coverage effects start timestamp	text (datetime_seconds_mdy)<br>Field Annotation: @HIDDEN-PDF @NOW @HIDDEN
	225	[glce_start_date]	GLP losing coverage effects start date	text (date_mdy)<br>Field Annotation: @HIDDEN-PDF @TODAY @HIDDEN
	226	[glce_header]	Effects of Prescription Coverage Changes	descriptive
	227	[glce_progress_bar]	65% Complete	descriptive
	228	[glce_loc_learn]	How did you first learn UT System prescription benefits would withdraw coverage for Saxenda, Wegovy, and Zepbound on September 1st, 2023?	radio, Required<br>1 My pharmacy when I tried to refill it<br>2 A letter from Express Scripts<br>3 An e-mail from UT System Benefits/Human Resources<br>4 I read about it in the Benefits Handbook<br>5 I was told by my Weight Wellness provider<br>6 I was told by my PCP or other specialist<br>7 I was told by a friend or colleague<br>98 Other
	229	[glce_loc_rxn]	What was you reaction to this news?[Select all that apply.]	checkbox, Required<br>1 glce_loc_rxn___1 Happy<br>2 glce_loc_rxn___2 Surprised<br>3 glce_loc_rxn___3 Angry or frustrated<br>4 glce_loc_rxn___4 Depressed<br>5 glce_loc_rxn___5 Anxious<br>6 glce_loc_rxn___6 Hopeless

			0 glce_loc_rxn___0 Indifferent<br><br>Field Annotation: @NONEOFTHEABOVE = '0'
230	[glce_form]	Section Header: *Effects of losing GLP-1 coverage for weight management...*<br>What was your weight on September 1st, 2023? It has been difficult to maintain my weight since GLP-1 coverage was withdrawn: {glce_wt_sep2023} lbs {glce_maint_wt} Has losing coverage for GLP-1 medications influenced your relationship with your current employer? {glce_emp_relation}	descriptive
231	[glce_wt_sep2023]<br><br>Show the field ONLY if:<br>[wmh_meds_12m(2)] = '1' OR [wmh_meds_12m(3)] = '1' OR [wmh_meds_current(2)] = '1' OR [wmh_meds_current(3)] = '1'	What was your weight on September 1st, 2023?<br>*lbs*	text (integer, Min: 100, Max: 1000), Required<br>Custom alignment: RH
232	[glce_maint_wt]	It has been difficult to maintain my weight since GLP-1 coverage was withdrawn.	radio (Matrix), Required<br>1 Strongly disagree<br>2 Disagree<br>3 Neutral<br>4 Agree<br>5 Strongly agree
233	[glce_emp_relation]<br><br>Show the field ONLY if:<br>([wmh_meds_12m(1)] = '1' OR [wmh_meds_12m(2)] = '1' OR [wmh_meds_12m(3)] = '1') OR ([wmh_meds_current(1)] = '1' OR [wmh_meds_current(2)] = '1' OR [wmh_meds_current(3)] = '1')	Has losing coverage for GLP-1 medications influenced your relationship with your current employer?	radio, Required<br>1 Yes, I feel very disappointed, and I am considering alternative jobs that offer GLP-1 coverage<br>2 Yes, I feel disappointed, but it does not influence my decision to continue working with my current employer<br>3 No, it does not influence the relationship with my employer at all<br><br>Custom alignment: LV
234	[glce_job_prod]	Section Header: *Since losing coverage for GLP-1...*<br>I am less productive at work.	radio (Matrix), Required<br>1 Strongly disagree<br>2 Disagree<br>3 Neutral<br>4 Agree<br>5 Strongly agree
235	[glce_job_absent]	I am absent more frequently from work.	radio (Matrix), Required<br>1 Strongly disagree<br>2 Disagree<br>3 Neutral<br>4 Agree<br>5 Strongly agree

	236	[glce_job_motivate]	I am less motivated to work.	radio (Matrix), Required<br>1 Strongly disagree<br>2 Disagree<br>3 Neutral<br>4 Agree<br>5 Strongly agree
	237	[glce_job_satisfy]	I am less satisfied with my job.	radio (Matrix), Required<br>1 Strongly disagree<br>2 Disagree<br>3 Neutral<br>4 Agree<br>5 Strongly agree
	238	[glce_job_value_emp]	I feel less valued by my employer.	radio (Matrix), Required<br>1 Strongly disagree<br>2 Disagree<br>3 Neutral<br>4 Agree<br>5 Strongly agree
	239	[glce_job_value_hlth]	I feel my employer does not value my health.	radio (Matrix), Required<br>1 Strongly disagree<br>2 Disagree<br>3 Neutral<br>4 Agree<br>5 Strongly agree
	240	[glce_burn_out]	I feel burned-out from my work.	radio (Matrix), Required<br>0 Never<br>1 A few times a year or less<br>2 Once a month or less<br>3 Once a week<br>4 A few times a week<br>5 Every day
	241	[glp_losing_coverage_effects_complete]	Section Header: *Form Status*<br>Complete?	dropdown<br>0 Incomplete<br>1 Unverified<br>2 Complete
Instrument: **GLP Professional Effects** (glp_professional_effects) **Enabled as survey**				
	242	[gpe_start_ts]	GLP professional effects start timestamp	text (datetime_seconds_mdy)<br>Field Annotation: @HIDDEN-PDF @NOW @HIDDEN
	243	[gpe_start_date]	GLP professional effects date	text (date_mdy)<br>Field Annotation: @HIDDEN-PDF @TODAY @HIDDEN

	244	[gpe_header]	Professional Effects with Loss of GLP-1 Coverage	descriptive
	245	[gpe_progress_bar]	70% Complete	descriptive
	246	[gpe_ppl_work_equal]	Section Header: *How do you feel people with obesity are treated in the workplace?*<br>I believe people with obesity are treated equally in the workplace.	radio (Matrix), Required<br>1 Yes<br>0 No
	247	[gpe_ppl_work_pay]	I believe people with obesity receive equal pay in the workplace.	radio (Matrix), Required<br>1 Yes<br>0 No
	248	[gpe_ppl_work_ews]	I have experienced discrimination in the workplace because of my weight.	radio (Matrix), Required<br>1 Yes<br>0 No
	249	[gpe_self_work_hap_sal]	Section Header: *How have you been treated in the workplace?*<br>I am happy with my current salary.	radio (Matrix), Required<br>1 Strongly disagree<br>2 Disagree<br>3 Neutral<br>4 Agree<br>5 Strongly agree
	250	[gpe_self_work_vary_sal]	Salaries vary significantly among employees at the same level in my workplace.	radio (Matrix), Required<br>1 Strongly disagree<br>2 Disagree<br>3 Neutral<br>4 Agree<br>5 Strongly agree
	251	[gpe_self_work_promo]	I feel confident going for a promotion.	radio (Matrix), Required<br>1 Strongly disagree<br>2 Disagree<br>3 Neutral<br>4 Agree<br>5 Strongly agree
	252	[gpe_self_work_advance]	I have career advancement opportunities in my workplace.	radio (Matrix), Required<br>1 Strongly disagree<br>2 Disagree<br>3 Neutral<br>4 Agree<br>5 Strongly agree
	253	[gpe_self_work_learn]	I learn new things and widen my experience in my workplace.	radio (Matrix), Required<br>1 Strongly disagree<br>2 Disagree<br>3 Neutral<br>4 Agree<br>5 Strongly agree

	254	[gpe_stigma_supervisor]	Section Header: *Prior to starting GLP-1 therapy...*<br>When delegating tasks and assignments, my supervisors considered my body weight.	radio (Matrix), Required<br>1 Strongly disagree<br>2 Disagree<br>3 Neutral<br>4 Agree<br>5 Strongly agree
	255	[gpe_stigma_profession]	My profession was influenced by my body weight.	radio (Matrix), Required<br>1 Strongly disagree<br>2 Disagree<br>3 Neutral<br>4 Agree<br>5 Strongly agree
	256	[gpe_stigma_cowork]	Coworkers treated me differently because of my body weight.	radio (Matrix), Required<br>1 Strongly disagree<br>2 Disagree<br>3 Neutral<br>4 Agree<br>5 Strongly agree
	257	[gpe_stigma_denied]	I had been unfairly denied promotions or salary increases in my workplace because of my body weight.	radio (Matrix), Required<br>1 Strongly disagree<br>2 Disagree<br>3 Neutral<br>4 Agree<br>5 Strongly agree
	258	[gpe_low_supervisor]	Section Header: *As a result of weight loss from GLP-1 therapy...*<br>When delegating tasks and assignment, my supervisor considers my lower body weight.	radio (Matrix), Required<br>1 Strongly disagree<br>2 Disagree<br>3 Neutral<br>4 Agree<br>5 Strongly agree
	259	[gpe_low_profession]	My profession is influenced by my lower body weight.	radio (Matrix), Required<br>1 Strongly disagree<br>2 Disagree<br>3 Neutral<br>4 Agree<br>5 Strongly agree
	260	[gpe_low_cowork]	Coworkers treat me differently because of my lower body weight.	radio (Matrix), Required<br>1 Strongly disagree<br>2 Disagree<br>3 Neutral<br>4 Agree

				5 Strongly agree
	261	[gpe_low_denied]	I have been unfairly denied promotions or salary increases in my workplace because of my lower body weight.	radio (Matrix), Required<br>1 Strongly disagree<br>2 Disagree<br>3 Neutral<br>4 Agree<br>5 Strongly agree
	262	[glp_professional_effects_complete]	Section Header: *Form Status*<br>Complete?	dropdown<br>0 Incomplete<br>1 Unverified<br>2 Complete
Instrument: **AOM Usage** (aom_usage) **Enabled as survey**				
	263	[au_start_ts]	AOM usage start timestamp	text (datetime_seconds_mdy)<br>Field Annotation: @HIDDEN-PDF @NOW @HIDDEN
	264	[au_start_date]	AOM usage start date	text (date_mdy)<br>Field Annotation: @HIDDEN-PDF @TODAY @HIDDEN
	265	[au_header]	Weight loss Medication Usage	descriptive
	266	[au_progress_bar]	75% Complete	descriptive
	267	[au_impact_qol]	Section Header: *How has being on a weight loss medication impacted your life?*<br>Weight loss medications improved my quality of life.	radio (Matrix), Required<br>1 Strongly disagree<br>2 Disagree<br>3 Neutral<br>4 Agree<br>5 Strongly agree
	268	[au_impact_control]	For the first time, I felt in control of my weight and health.	radio (Matrix), Required<br>1 Strongly disagree<br>2 Disagree<br>3 Neutral<br>4 Agree<br>5 Strongly agree
	269	[au_imp_confident]	Section Header: *As my weight and health improved while taking weight loss medications...*<br>I felt more confident.	radio (Matrix), Required<br>1 Strongly disagree<br>2 Disagree<br>3 Neutral<br>4 Agree<br>5 Strongly agree
	270	[au_imp_serious]	People take me more seriously.	radio (Matrix), Required<br>1 Strongly disagree<br>2 Disagree<br>3 Neutral

			4 Agree<br>5 Strongly agree
271	[au_imp_worth]	People started looking past my body size and saw my worth.	radio (Matrix), Required<br>1 Strongly disagree<br>2 Disagree<br>3 Neutral<br>4 Agree<br>5 Strongly agree
272	[au_imp_social]	I felt less distress in social interactions.	radio (Matrix), Required<br>1 Strongly disagree<br>2 Disagree<br>3 Neutral<br>4 Agree<br>5 Strongly agree
273	[au_imp_relationship]	I felt less distress in relationship interactions (e.g., with partner or in dating).	radio (Matrix), Required<br>1 Strongly disagree<br>2 Disagree<br>3 Neutral<br>4 Agree<br>5 Strongly agree
274	[au_imp_professional]	I felt less distress in professional interactions.	radio (Matrix), Required<br>1 Strongly disagree<br>2 Disagree<br>3 Neutral<br>4 Agree<br>5 Strongly agree
275	[au_imp_career]	I was able to advance in my career with fewer barriers.	radio (Matrix), Required<br>1 Strongly disagree<br>2 Disagree<br>3 Neutral<br>4 Agree<br>5 Strongly agree
276	[aom_usage_complete]	Section Header: *Form Status*<br>Complete?	dropdown<br>0 Incomplete<br>1 Unverified<br>2 Complete
Instrument: **AOM Weight Loss Attempts** (aom_weight_loss_attempts)			**Enabled as survey**
277	[awa_start_ts]	AOM weight loss attempts start timestamp	text (datetime_seconds_mdy)<br>Field Annotation: @HIDDEN-PDF @NOW @HIDDEN
278	[awa_start_date]	AOM weight loss attempts start date	text (date_mdy)<br>Field Annotation: @HIDDEN-PDF @TODAY

				@HIDDEN
	279	[awa_header]	Weight Loss Attempts With Anti-obesity Medication	descriptive
	280	[awa_progress_bar]	80% Complete	descriptive
	281	[awa_consult_hcp]	Section Header: *Prior to enrolling in the UT Southwestern Weight Wellness Program...*<br>Which of the following had you consulted for weight loss?[Select all that apply.]	checkbox, Required<br>1 | awa_consult_hcp___1 | Primary care provider<br>2 | awa_consult_hcp___2 | Obesity medicine specialist<br>3 | awa_consult_hcp___3 | Other medicine specialist (e.g. cardiologist)<br>4 | awa_consult_hcp___4 | Bariatric surgeon<br>5 | awa_consult_hcp___5 | Dietitian<br>6 | awa_consult_hcp___6 | Exercise specialist/Persona trainer<br>98 | awa_consult_hcp___98 | Other<br>0 | awa_consult_hcp___0 | None<br><br>Field Annotation: @NONEOFTHEABOVE = '0'
	282	[awa_tried]	Section Header: *Prior to any weight loss medications, which of thefollowing had you tried?*<br>* must provide value Diets Phone Applications "Apps" Commercial Weight Loss Programs Online Weight Loss Platforms {awa_tried_diet} {awa_tried_app} {awa_tried_commercial} {awa_tried_online} Home Delivery Meal Prep Kits (e.g. Hello Fresh, Blue Apron) Prepared Meals (e.g. Factor, Snap Kitchen, Jenny Craig) Online counseling or coaching (e.g. Berry Street, Nourish, Atwell) {awa_tried_kit} {awa_tried_meal} {awa_tried_coach}	descriptive
	283	[awa_tried_diet]	Prior to any weight loss medications, which of the following had you tried: Diets[Select all that apply.]	checkbox, Required<br>0 | awa_tried_diet___0 | None<br>1 | awa_tried_diet___1 | Low calorie<br>2 | awa_tried_diet___2 | Low fat<br>3 | awa_tried_diet___3 | Low carb<br>4 | awa_tried_diet___4 | Keto<br>5 | awa_tried_diet___5 | Vegetarian/Vegan<br>6 | awa_tried_diet___6 | Mediterranean<br>7 | awa_tried_diet___7 | Whole 30<br>8 | awa_tried_diet___8 | Paleo<br>9 | awa_tried_diet___9 | Intermittent fasting<br>98 | awa_tried_diet___98 | Other<br><br>Custom alignment: LV<br>Field Annotation: @NONEOFTHEABOVE = '0'

	284	[awa_tried_app]	Prior to any weight loss medications, which of the following had you tried: Phone Applications "Apps"[Select all that apply.]	checkbox, Required<br>0	awa_tried_app___0	None<br>1	awa_tried_app___1	Calorie King<br>2	awa_tried_app___2	Cronometer<br>3	awa_tried_app___3	FatSecret<br>4	awa_tried_app___4	Fooducate<br>5	awa_tried_app___5	Lose It!<br>6	awa_tried_app___6	MyFitness Pal<br>7	awa_tried_app___7	MyNet Diary<br>8	awa_tried_app___8	Noom<br>9	awa_tried_app___9	Yazio<br>98	awa_tried_app___98	Other<br><br>Custom alignment: LV<br>Field Annotation: @NONEOFTHEABOVE = '0'
	285	[awa_tried_commercial]	Prior to any weight loss medications, which of the following had you tried: Commercial Weight Loss Programs[Select all that apply.]	checkbox, Required<br>0	awa_tried_commercial___0	None<br>1	awa_tried_commercial___1	Weight Watchers<br>2	awa_tried_commercial___2	Jenny Craig<br>3	awa_tried_commercial___3	NutriSystem<br>4	awa_tried_commercial___4	South Beach<br>5	awa_tried_commercial___5	Optifast<br>6	awa_tried_commercial___6	Ideal Protein<br>7	awa_tried_commercial___7	Huel<br>8	awa_tried_commercial___8	HMR<br>9	awa_tried_commercial___9	Slim Fast<br>98	awa_tried_commercial___98	Other<br><br>Custom alignment: LV<br>Field Annotation: @NONEOFTHEABOVE = '0'
	286	[awa_tried_kit]	Prior to any weight loss medications, have you tried: Home Delivery Meal Prep Kit	yesno, Required<br>1	Yes<br>0	No<br><br>Custom alignment: LV
	287	[awa_tried_meal]	Prior to any weight loss medications, have you tried: Prepared Meals	yesno, Required<br>1	Yes<br>0	No<br><br>Custom alignment: LV
	288	[awa_tried_online]	Prior to any weight loss medications, which of the following had you tried: Online weight loss platform[Select all that apply.]	checkbox, Required<br>0	awa_tried_online___0	None

<table>
<tr><td></td><td></td><td></td><td></td><td>
<table>
<tr><td>1</td><td>awa_tried_online___1</td><td>Noom</td></tr>
<tr><td>2</td><td>awa_tried_online___2</td><td>Virta</td></tr>
<tr><td>3</td><td>awa_tried_online___3</td><td>Calibrate</td></tr>
<tr><td>4</td><td>awa_tried_online___4</td><td>Roman/Ro</td></tr>
<tr><td>5</td><td>awa_tried_online___5</td><td>Weight Watchers</td></tr>
<tr><td>6</td><td>awa_tried_online___6</td><td>Found</td></tr>
<tr><td>7</td><td>awa_tried_online___7</td><td>Wondr</td></tr>
<tr><td>8</td><td>awa_tried_online___8</td><td>Naturally Slim</td></tr>
<tr><td>9</td><td>awa_tried_online___9</td><td>Slim for Life</td></tr>
<tr><td>10</td><td>awa_tried_online___10</td><td>Omada</td></tr>
<tr><td>98</td><td>awa_tried_online___98</td><td>Other</td></tr>
</table>
Custom alignment: LV
Field Annotation: @NONEOFTHEABOVE = '0'</td></tr>
<tr><td></td><td>289</td><td>[awa_tried_online_rx]

Show the field ONLY if: [awa_tried_online(1)] = '1' OR [awa_tried_online(2)] = '1' OR [awa_tried_online(3)] = '1' OR [awa_tried_online(4)] = '1' OR [awa_tried_online(5)] = '1' OR [awa_tried_online(6)] = '1' OR [awa_tried_online(7)] = '1' OR [awa_tried_online(8)] = '1' OR [awa_tried_online(9)] = '1' OR [awa_tried_online(10)] = '1' OR [awa_tried_online(98)] = '1'</td><td>Did you receive a weight loss medication from [awa_tried_online:checked]?</td><td>yesno, Required
<table>
<tr><td>1</td><td>Yes</td></tr>
<tr><td>0</td><td>No</td></tr>
</table></td></tr>
<tr><td></td><td>290</td><td>[awa_tried_coach]</td><td>Prior to any weight loss medications, have you tried: Online counseling or coaching for weight loss?</td><td>yesno, Required
<table>
<tr><td>1</td><td>Yes</td></tr>
<tr><td>0</td><td>No</td></tr>
</table>
Custom alignment: LV</td></tr>
<tr><td></td><td>291</td><td>[awa_sug]</td><td>Section Header: Which of the following, if any, would you say should be used while taking weight loss medications?

* must provide value Diets Phone Applications "Apps" Commercial Weight Loss Programs Online Weight Loss Platforms {awa_sug_diet} {awa_sug_app} {awa_sug_commercial} {awa_sug_online} Home Delivery Meal Prep Kits (e.g. Hello Fresh, Blue Apron) Prepared Meals (e.g. Factor, Snap Kitchen, Jenny Craig) Online counseling or coaching (e.g. Berry Street, Nourish, Atwell) {awa_sug_kit} {awa_sug_meal} {awa_sug_coach}</td><td>descriptive</td></tr>
<tr><td></td><td>292</td><td>[awa_sug_diet]</td><td>Which of the following, if any, would you say should be used while taking weight loss medications: Diets[Select all that apply.]</td><td>checkbox, Required
<table>
<tr><td>0</td><td>awa_sug_diet___0</td><td>None</td></tr>
<tr><td>1</td><td>awa_sug_diet___1</td><td>Low calorie</td></tr>
</table></td></tr>
</table>

2	awa_sug_diet___2	Low fat
3	awa_sug_diet___3	Low carb
4	awa_sug_diet___4	Keto
5	awa_sug_diet___5	Vegetarian/Vegan
6	awa_sug_diet___6	Mediterranean
7	awa_sug_diet___7	Whole 30
8	awa_sug_diet___8	Paleo
9	awa_sug_diet___9	Intermittent fasting
98	awa_sug_diet___98	Other

Custom alignment: LV
Field Annotation: @NONEOFTHEABOVE = '0'

	293	[awa_sug_app]	Which of the following, if any, would you say should be used while taking weight loss medications: Phone Applications "Apps"[Select all that apply.]	checkbox, Required

0	awa_sug_app___0	None
1	awa_sug_app___1	Calorie King
2	awa_sug_app___2	Cronometer
3	awa_sug_app___3	FatSecret
4	awa_sug_app___4	Fooducate
5	awa_sug_app___5	Lose It!
6	awa_sug_app___6	MyFitness Pal
7	awa_sug_app___7	MyNet Diary
8	awa_sug_app___8	Noom
9	awa_sug_app___9	Yazio
98	awa_sug_app___98	Other

Custom alignment: LV
Field Annotation: @NONEOFTHEABOVE = '0'

	294	[awa_sug_commercial]	Which of the following, if any, would you say should be used while taking weight loss medications: Commercial Weight Loss Programs[Select all that apply.]	checkbox, Required

0	awa_sug_commercial___0	None
1	awa_sug_commercial___1	Weight Watchers
2	awa_sug_commercial___2	Jenny Craig
3	awa_sug_commercial___3	NutriSystem
4	awa_sug_commercial___4	South Beach
5	awa_sug_commercial___5	Optifast
6	awa_sug_commercial___6	Ideal Protein
7	awa_sug_commercial___7	Huel
8	awa_sug_commercial___8	HMR
9	awa_sug_commercial___9	Slim Fast
98	awa_sug_commercial___98	Other

Custom alignment: LV

				Field Annotation: @NONEOFTHEABOVE = '0'
	295	[awa_sug_kit]	Should the following be used while taking weight loss medications: Home Delivery Meal Prep Kit	yesno, Required<br>1 Yes<br>0 No<br><br>Custom alignment: LV
	296	[awa_sug_meal]	Should the following be used while taking weight loss medications: Prepared Meals	yesno, Required<br>1 Yes<br>0 No<br><br>Custom alignment: LV
	297	[awa_sug_online]	Which of the following, if any, would you say should be used while taking weight loss medications: Online weight loss platform?[Select all that apply.]	checkbox, Required<br>0 awa_sug_online___0 None<br>1 awa_sug_online___1 Noom<br>2 awa_sug_online___2 Virta<br>3 awa_sug_online___3 Calibrate<br>4 awa_sug_online___4 Roman/Ro<br>5 awa_sug_online___5 Weight Watchers<br>6 awa_sug_online___6 Found<br>7 awa_sug_online___7 Wondr<br>8 awa_sug_online___8 Naturally Slim<br>9 awa_sug_online___9 Slim for Life<br>10 awa_sug_online___10 Omada<br>98 awa_sug_online___98 Other<br><br>Custom alignment: LV<br>Field Annotation: @NONEOFTHEABOVE = '0'
	298	[awa_sug_coach]	Should the following be used while taking weight loss medications: Online counseling or coaching for weight loss	yesno, Required<br>1 Yes<br>0 No<br><br>Custom alignment: LV
	299	[awa_rplc]	Section Header: *In your opinion, which of the following are adequate replacements for weight loss medications?*<br>* must provide value Diets Phone Applications "Apps" Commercial Weight Loss Programs Online Weight Loss Platforms {awa_rplc_diet} {awa_rplc_app} {awa_rplc_commercial} {awa_rplc_online} Home Delivery Meal Prep Kits (e.g. Hello Fresh, Blue Apron) Prepared Meals (e.g. Factor, Snap Kitchen, Jenny Craig) Online counseling or coaching (e.g. Berry Street, Nourish, Atwell) {awa_rplc_kit} {awa_rplc_meal} {awa_rplc_coach}	descriptive
	300	[awa_rplc_diet]	In your opinion, which of the following are adequate replacements for weight loss medications: Diets[Select all that apply.]	checkbox, Required<br>0 awa_rplc_diet___0 None<br>1 awa_rplc_diet___1 Low calorie

				2 | awa_rplc_diet___2 | Low fat<br>3 | awa_rplc_diet___3 | Low carb<br>4 | awa_rplc_diet___4 | Keto<br>5 | awa_rplc_diet___5 | Vegetarian/Vegan<br>6 | awa_rplc_diet___6 | Mediterranean<br>7 | awa_rplc_diet___7 | Whole 30<br>8 | awa_rplc_diet___8 | Paleo<br>9 | awa_rplc_diet___9 | Intermittent fasting<br>98 | awa_rplc_diet___98 | Other<br><br>Custom alignment: LV<br>Field Annotation: @NONEOFTHEABOVE = '0'
	301	[awa_rplc_app]	In your opinion, which of the following are adequate replacements for weight loss medications: Phone Applications "Apps"[Select all that apply.]	checkbox, Required<br>0 | awa_rplc_app___0 | None<br>1 | awa_rplc_app___1 | Calorie King<br>2 | awa_rplc_app___2 | Cronometer<br>3 | awa_rplc_app___3 | FatSecret<br>4 | awa_rplc_app___4 | Fooducate<br>5 | awa_rplc_app___5 | Lose It!<br>6 | awa_rplc_app___6 | MyFitness Pal<br>7 | awa_rplc_app___7 | MyNet Diary<br>8 | awa_rplc_app___8 | Noom<br>9 | awa_rplc_app___9 | Yazio<br>98 | awa_rplc_app___98 | Other<br><br>Custom alignment: LV<br>Field Annotation: @NONEOFTHEABOVE = '0'
	302	[awa_rplc_commercial]	In your opinion, which of the following are adequate replacements for weight loss medications: Commercial Weight Loss Programs[Select all that apply.]	checkbox, Required<br>0 | awa_rplc_commercial___0 | None<br>1 | awa_rplc_commercial___1 | Weight Watchers<br>2 | awa_rplc_commercial___2 | Jenny Craig<br>3 | awa_rplc_commercial___3 | NutriSystem<br>4 | awa_rplc_commercial___4 | South Beach<br>5 | awa_rplc_commercial___5 | Optifast<br>6 | awa_rplc_commercial___6 | Ideal Protein<br>7 | awa_rplc_commercial___7 | Huel<br>8 | awa_rplc_commercial___8 | HMR<br>9 | awa_rplc_commercial___9 | Slim Fast<br>98 | awa_rplc_commercial___98 | Other<br><br>Custom alignment: LV

				Field Annotation: @NONEOFTHEABOVE = '0'
	303	[awa_rplc_online]	In your opinion, which of the following are adequate replacements for weight loss medications: Online weight loss platform[Select all that apply.]	checkbox, Required<br>0 | awa_rplc_online___0 | None<br>1 | awa_rplc_online___1 | Noom<br>2 | awa_rplc_online___2 | Virta<br>3 | awa_rplc_online___3 | Calibrate<br>4 | awa_rplc_online___4 | Roman/Ro<br>5 | awa_rplc_online___5 | Weight Watchers<br>6 | awa_rplc_online___6 | Found<br>7 | awa_rplc_online___7 | Wondr<br>8 | awa_rplc_online___8 | Naturally Slim<br>9 | awa_rplc_online___9 | Slim for Life<br>10 | awa_rplc_online___10 | Omada<br>98 | awa_rplc_online___98 | Other<br><br>Custom alignment: LV<br>Field Annotation: @NONEOFTHEABOVE = '0'
	304	[awa_rplc_kit]	Are the following adequate replacements for weight loss medications: Home Delivery Meal Prep Kit	yesno, Required<br>1 | Yes<br>0 | No<br><br>Custom alignment: LV
	305	[awa_rplc_meal]	Are the following adequate replacements for weight loss medications: Prepared Meals	yesno, Required<br>1 | Yes<br>0 | No<br><br>Custom alignment: LV
	306	[awa_rplc_coach]	Are the following adequate replacements for weight loss medications: Online counseling or coaching for weight loss	yesno, Required<br>1 | Yes<br>0 | No<br><br>Custom alignment: LV
	307	[aom_weight_loss_attempts_complete]	Section Header: *Form Status*<br>Complete?	dropdown<br>0 | Incomplete<br>1 | Unverified<br>2 | Complete
**Instrument: Non Rx Weight Loss Attempts** (non_rx_weight_loss_attempts) **Enabled as survey**				
	308	[nrxwa_start_ts]	No prescription weight loss attempts start timestamp	text (datetime_seconds_mdy)<br>Field Annotation: @HIDDEN-PDF @NOW @HIDDEN
	309	[nrxwa_start_date]	No prescription weight loss attempts start date	text (date_mdy)<br>Field Annotation: @HIDDEN-PDF @TODAY @HIDDEN
	310	[nrxwa_header]	Weight Loss Attempts Without Medication	descriptive

	311	[nrxwa_progress_bar]	85% Complete	descriptive
	312	[nrxwa_consult_hcp]	Section Header: *Prior to enrolling in the UT Southwestern Weight Wellness Program...*<br><br>Which of the following had you consulted for weight loss?[Select all that apply.]	checkbox, Required<br>1 | nrxwa_consult_hcp___1 | Primary care provider<br>2 | nrxwa_consult_hcp___2 | Obesity medicin specialist<br>3 | nrxwa_consult_hcp___3 | Other medicine specialist (e.g. cardiologist)<br>4 | nrxwa_consult_hcp___4 | Bariatric surgeo<br>5 | nrxwa_consult_hcp___5 | Dietitian<br>6 | nrxwa_consult_hcp___6 | Exercise specialist/Perso trainer<br>98 | nrxwa_consult_hcp___98 | Other<br>0 | nrxwa_consult_hcp___0 | None<br><br>Field Annotation: @NONEOFTHEABOVE = '0'
	313	[nrxwa_tried]	Section Header: *Which of thefollowing had you tried?*<br><br>* must provide value Diets Phone Applications "Apps" Commercial Weight Loss Programs Online Weight Loss Platforms {nrxwa_tried_diet} {nrxwa_tried_app} {nrxwa_tried_commercial} {nrxwa_tried_online} Home Delivery Meal Prep Kits (e.g. Hello Fresh, Blue Apron) Prepared Meals (e.g. Factor, Snap Kitchen, Jenny Craig) Online counseling or coaching (e.g. Berry Street, Nourish, Atwell) {nrxwa_tried_kit} {nrxwa_tried_meal} {nrxwa_tried_coach}	descriptive
	314	[nrxwa_tried_diet]	Which of the following had you tried: Diets[Select all that apply.]	checkbox, Required<br>0 | nrxwa_tried_diet___0 | None<br>1 | nrxwa_tried_diet___1 | Low calorie<br>2 | nrxwa_tried_diet___2 | Low fat<br>3 | nrxwa_tried_diet___3 | Low carb<br>4 | nrxwa_tried_diet___4 | Keto<br>5 | nrxwa_tried_diet___5 | Vegetarian/Vegan<br>6 | nrxwa_tried_diet___6 | Mediterranean<br>7 | nrxwa_tried_diet___7 | Whole 30<br>8 | nrxwa_tried_diet___8 | Paleo<br>9 | nrxwa_tried_diet___9 | Intermittent fasting<br>98 | nrxwa_tried_diet___98 | Other<br><br>Custom alignment: LV<br>Field Annotation: @NONEOFTHEABOVE = '0'
	315	[nrxwa_tried_app]	Which of the following had you tried: Phone Applications "Apps"[Select all that apply.]	checkbox, Required<br>0 | nrxwa_tried_app___0 | None<br>1 | nrxwa_tried_app___1 | Calorie King

				2 | nrxwa_tried_app___2 | Cronometer<br>3 | nrxwa_tried_app___3 | FatSecret<br>4 | nrxwa_tried_app___4 | Fooducate<br>5 | nrxwa_tried_app___5 | Lose It!<br>6 | nrxwa_tried_app___6 | MyFitness Pal<br>7 | nrxwa_tried_app___7 | MyNet Diary<br>8 | nrxwa_tried_app___8 | Noom<br>9 | nrxwa_tried_app___9 | Yazio<br>98 | nrxwa_tried_app___98 | Other<br><br>Custom alignment: LV<br>Field Annotation: @NONEOFTHEABOVE = '0'
	316	[nrxwa_tried_commercial]	Which of the following had you tried: Commercial Weight Loss Programs[Select all that apply.]	checkbox, Required<br>0 | nrxwa_tried_commercial___0 | None<br>1 | nrxwa_tried_commercial___1 | Weight Watchers<br>2 | nrxwa_tried_commercial___2 | Jenny Craig<br>3 | nrxwa_tried_commercial___3 | NutriSyste[illegible]<br>4 | nrxwa_tried_commercial___4 | South Beach<br>5 | nrxwa_tried_commercial___5 | Optifast<br>6 | nrxwa_tried_commercial___6 | Ideal Protein<br>7 | nrxwa_tried_commercial___7 | Huel<br>8 | nrxwa_tried_commercial___8 | HMR<br>9 | nrxwa_tried_commercial___9 | Slim Fast<br>98 | nrxwa_tried_commercial___98 | Other<br><br>Custom alignment: LV<br>Field Annotation: @NONEOFTHEABOVE = '0'
	317	[nrxwa_tried_kit]	Have you tried: Home Delivery Meal Prep Kit	yesno, Required<br>1 | Yes<br>0 | No<br><br>Custom alignment: LV
	318	[nrxwa_tried_meal]	Have you tried: Prepared Meals	yesno, Required<br>1 | Yes<br>0 | No<br><br>Custom alignment: LV
	319	[nrxwa_tried_online]	Which of the following had you tried: Online weight loss platform[Select all that apply.]	checkbox, Required<br>0 | nrxwa_tried_online___0 | None<br>1 | nrxwa_tried_online___1 | Noom<br>2 | nrxwa_tried_online___2 | Virta<br>3 | nrxwa_tried_online___3 | Calibrate

				4 | nrxwa_tried_online___4 | Roman/Ro<br>5 | nrxwa_tried_online___5 | Weight Watchers<br>6 | nrxwa_tried_online___6 | Found<br>7 | nrxwa_tried_online___7 | Wondr<br>8 | nrxwa_tried_online___8 | Naturally Slim<br>9 | nrxwa_tried_online___9 | Slim for Life<br>10 | nrxwa_tried_online___10 | Omada<br>98 | nrxwa_tried_online___98 | Other<br><br>Custom alignment: LV<br>Field Annotation: @NONEOFTHEABOVE = '0'
	320	[nrxwa_tried_coach]	Have you tried: Online counseling or coaching for weight loss	yesno, Required<br>1 | Yes<br>0 | No<br><br>Custom alignment: LV
	321	[non_rx_weight_loss_attempts_complete]	Section Header: *Form Status*<br>Complete?	dropdown<br>0 | Incomplete<br>1 | Unverified<br>2 | Complete
**Instrument: Non GLP Weight Loss Attempts Quantified (non_glp_weight_loss_attempts_quantified) Enabled as survey**				
	322	[ngwa_start_ts]	Non GLP weight loss attempts start timestamp	text (datetime_seconds_mdy)<br>Field Annotation: @HIDDEN-PDF @NOW @HIDDEN
	323	[ngwa_start_date]	Non GLP weight loss attempts start date	text (date_mdy)<br>Field Annotation: @HIDDEN-PDF @TODAY @HIDDEN
	324	[ngwa_header]	Non-GLP Weight Loss Attempts	descriptive
	325	[ngwa_progress_bar]	90% Complete	descriptive
	326	[ngwa_wt_lost_max_lbs]	Section Header: *In your lifetime...*<br>What is the most weight you have ever lost?<br>*lbs*	text (integer, Min: 0, Max: 1000), Required
	327	[ngwa_wt_lost_mode]	Which was the most helpful for weight loss?	radio, Required<br>1 | Weight loss medication(s)<br>2 | Weight loss surgery (bariatric surgery)<br>3 | Dieting<br>4 | Exercise<br>98 | Other<br>0 | None<br><br>Custom alignment: LV
	328	[preaom_wl_20]<br><br>Show the field ONLY if:	Section Header: *How many times in your life have you lost...*<br>...20 lb. or more?<br>*times*	text (integer, Min: 0, Max: 99), Required<br>Field Annotation: @PLACEHOLDER = 'Provide an integer value'

		[ngwa_wt_lost_max_lbs] >= 20 AND ([ngwa_wt_lost_mode] = '1' OR [ngwa_wt_lost_mode] = '2' OR [ngwa_wt_lost_mode] = '3' OR [ngwa_wt_lost_mode] = '4' OR [ngwa_wt_lost_mode] = '98' OR [ngwa_wt_lost_mode] = '0')		
	329	[preaom_wl_35]<br>Show the field ONLY if: [ngwa_wt_lost_max_lbs] >= 35 AND ([ngwa_wt_lost_mode] = '1' OR [ngwa_wt_lost_mode] = '2' OR [ngwa_wt_lost_mode] = '3' OR [ngwa_wt_lost_mode] = '4' OR [ngwa_wt_lost_mode] = '98' OR [ngwa_wt_lost_mode] = '0')	...35 lb. or more?<br>*times*	text (integer, Min: 0, Max: 99), Required<br>Field Annotation: @PLACEHOLDER = 'Provide an integer value'
	330	[preaom_wl_50]<br>Show the field ONLY if: [ngwa_wt_lost_max_lbs] >= 50 AND ([ngwa_wt_lost_mode] = '1' OR [ngwa_wt_lost_mode] = '2' OR [ngwa_wt_lost_mode] = '3' OR [ngwa_wt_lost_mode] = '4' OR [ngwa_wt_lost_mode] = '98' OR [ngwa_wt_lost_mode] = '0')	...50 lb. or more?<br>*times*	text (integer, Min: 0, Max: 99), Required<br>Field Annotation: @PLACEHOLDER = 'Provide an integer value'
	331	[preaom_wl_100]<br>Show the field ONLY if: [ngwa_wt_lost_max_lbs] >= 100 AND ([ngwa_wt_lost_mode] = '1' OR [ngwa_wt_lost_mode] = '2' OR [ngwa_wt_lost_mode] = '3' OR [ngwa_wt_lost_mode] = '4' OR [ngwa_wt_lost_mode] = '98' OR [ngwa_wt_lost_mode] = '0')	...100 lb. or more?<br>*times*	text (integer, Min: 0, Max: 99), Required<br>Field Annotation: @PLACEHOLDER = 'Provide an integer value'
	332	[non_glp_weight_loss_attempts_quantified_complete]	Section Header: *Form Status*<br>Complete?	dropdown<br>0 Incomplete<br>1 Unverified<br>2 Complete

Supplemental Figure 1. GLP-1 Loss of Coverage Study Survey Codebook

Participants completed self-report items on demographics, weight-loss medication history, medical history, GLP-1 RA use, impacts of insurance coverage loss, professional life, general AOM use, and non-prescription weight-loss attempts. All items were developed by the research team, except for the validated Everyday Discrimination Scale (EDS).

Abbreviations: GLP-1 RA, glucagon-like peptide-1 receptor agonist; EDS, Everyday Discrimination Scale; AOM, anti-obesity medication.
